# Nervous System Regionalization Entails Axial Allocation before Neural Differentiation

**DOI:** 10.1016/j.cell.2018.09.040

**Published:** 2018-11-01

**Authors:** Vicki Metzis, Sebastian Steinhauser, Edvinas Pakanavicius, Mina Gouti, Despina Stamataki, Kenzo Ivanovitch, Thomas Watson, Teresa Rayon, S. Neda Mousavy Gharavy, Robin Lovell-Badge, Nicholas M. Luscombe, James Briscoe

**Affiliations:** 1 The Francis Crick Institute, London NW1 1AT, UK; 2 Max-Delbrück Center for Molecular Medicine, Berlin 13092, Germany; 3 UCL Genetics Institute, Department of Genetics Evolution and Environment, University College London, London WC1E 6BT, UK; 4 Okinawa Institute of Science and Technology Graduate University, Onna-son, Kunigami-gun, Okinawa 904-0495, Japan

**Keywords:** neural induction, ATAC-seq, CDX, embryonic development, chromatin, spinal cord, WNT signaling, stem cells and development, computational genomics, gene regulation

## Abstract

Neural induction in vertebrates generates a CNS that extends the rostral-caudal length of the body. The prevailing view is that neural cells are initially induced with anterior (forebrain) identity; caudalizing signals then convert a proportion to posterior fates (spinal cord). To test this model, we used chromatin accessibility to define how cells adopt region-specific neural fates. Together with genetic and biochemical perturbations, this identified a developmental time window in which genome-wide chromatin-remodeling events preconfigure epiblast cells for neural induction. Contrary to the established model, this revealed that cells commit to a regional identity before acquiring neural identity. This “primary regionalization” allocates cells to anterior or posterior regions of the nervous system, explaining how cranial and spinal neurons are generated at appropriate axial positions. These findings prompt a revision to models of neural induction and support the proposed dual evolutionary origin of the vertebrate CNS.

## Highlights

• Chromatin accessibility defines neural progenitor identity
• A limited developmental window exists to establish spinal cord competency
• Cells acquire axial identity prior to neural identity

## Graphical Abstract

**Figure:**
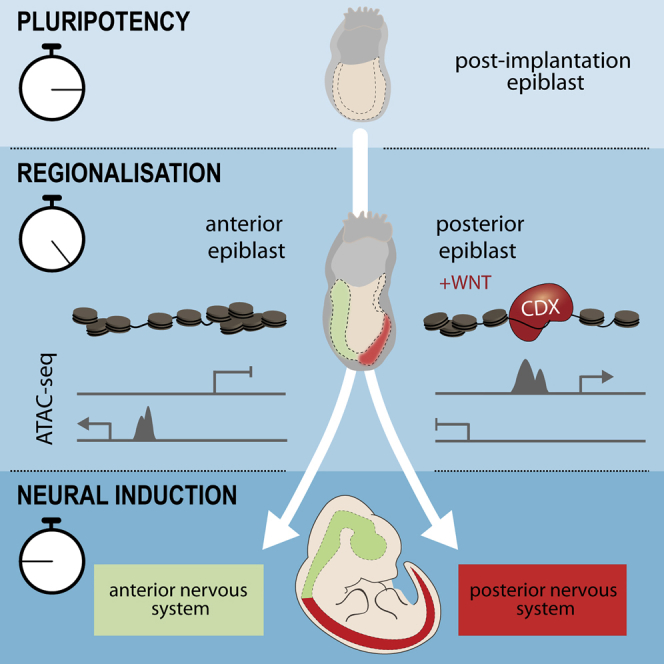

## Teaser

Regional identity precedes neural identity of the anterior and posterior nervous systems.

## Introduction

Development of the vertebrate nervous system begins at gastrulation and continues as the principal axis elongates, resulting in a nervous system extending along the anterior-posterior (AP) length of the body (Stern, 2006). The critical role of the organizer in specifying neural fate was established by the pioneering work of Spemann and Mangold (1924), demonstrating that transplantation of the organizer produced a secondary neural axis. Attention then turned to identifying the signals emanating from the organizer and understanding how different rostral-caudal regions of the nervous system are generated (Stern, 2001, Stern et al., 2006).

Several models have been proposed to explain rostral-caudal regionalization. Nieuwkoop (1952), building on Waddington (1940), proposed a two-step mechanism known as “activation-transformation.” This contends that cells first adopt a neural identity equivalent to the anterior nervous system (“activation”). “Transformation” then converts a proportion of these cranial-like cells to more caudal fates, such as midbrain, hindbrain, and spinal cord (Nieuwkoop and Nigtevecht, 1954, Stern, 2001). In this view, all neural cells are first specified with an anterior identity before they acquire more caudal fates. Whether this mechanism applies to all axial levels of the nervous system is unresolved; nevertheless, it remains the prevailing view (Stern, 2001, Stern, 2006).

The anterior nervous system in vertebrates (forebrain, midbrain, and hindbrain) is formed from cells in the anterior epiblast. By contrast, spinal cord cells are produced during axis elongation by axial stem cells known as neuromesodermal progenitors (NMPs). These bipotent cells arise in the caudal lateral epiblast and contribute to both spinal cord and somites (Tzouanacou et al., 2009). NMPs are exposed to fibroblast growth factor (FGF) and WNT signaling and are marked by the expression of transcription factors SOX2, *T/Brachyury*, and CDX1, 2, and 4 (Gouti et al., 2017, Tsakiridis et al., 2014). Deletion of *T/Bra*, *Cdx* genes, or reduced WNT signaling severely abrogates axis elongation, resulting in post-occipital loss of spinal cord and somites (Amin et al., 2016, Takada et al., 1994, Yamaguchi et al., 1999, Young et al., 2009). *In vitro*, timely pulses of FGF and WNT signals to mouse embryonic stem cells (ESCs) produces cells resembling NMPs (Gouti et al., 2017, Koch et al., 2017). These cells can be differentiated into spinal cord progenitors expressing cervical-thoracic *Hox* genes (Gouti et al., 2014, Tsakiridis et al., 2014). ESCs differentiated in the absence of WNT generate neural progenitors (NPs) with a caudal limit corresponding to the hindbrain and cervical spinal cord (Gouti et al., 2014). These observations appear to challenge the activation-transformation hypothesis and support older ideas that distinct mechanisms specify different regions of the nervous system (Mangold, 1933).

To determine the sequence of events that establish a regionalized nervous system, an unbiased definition of cell identity is required. Enhancer usage, determined by chromatin accessibility, has been used to define different cell types and has been shown to better resolve cell identity than gene expression (Corces et al., 2016). A repertoire of enhancers drives AP-specific expression of many neural genes throughout the nervous system (Epstein et al., 1999, Uchikawa et al., 2003). This suggests that enhancer usage can be used to define neural cell identity at different AP positions.

Here, we assay temporal changes in chromatin accessibility that occur in differentiating NPs with defined axial fates. We find that the competency to generate spinal cord is transient and dependent on chromatin remodeling events driven by CDX transcription factors (TFs). Contrary to the activation-transformation hypothesis, our data indicate that axial identity is established in cells before neural identity. These findings prompt a revision to models of neural induction and nervous system regionalization.

## Results

### *In Vitro* Generation of Anterior, Hindbrain, or Spinal Cord Neural Progenitors

**Figure 1:**
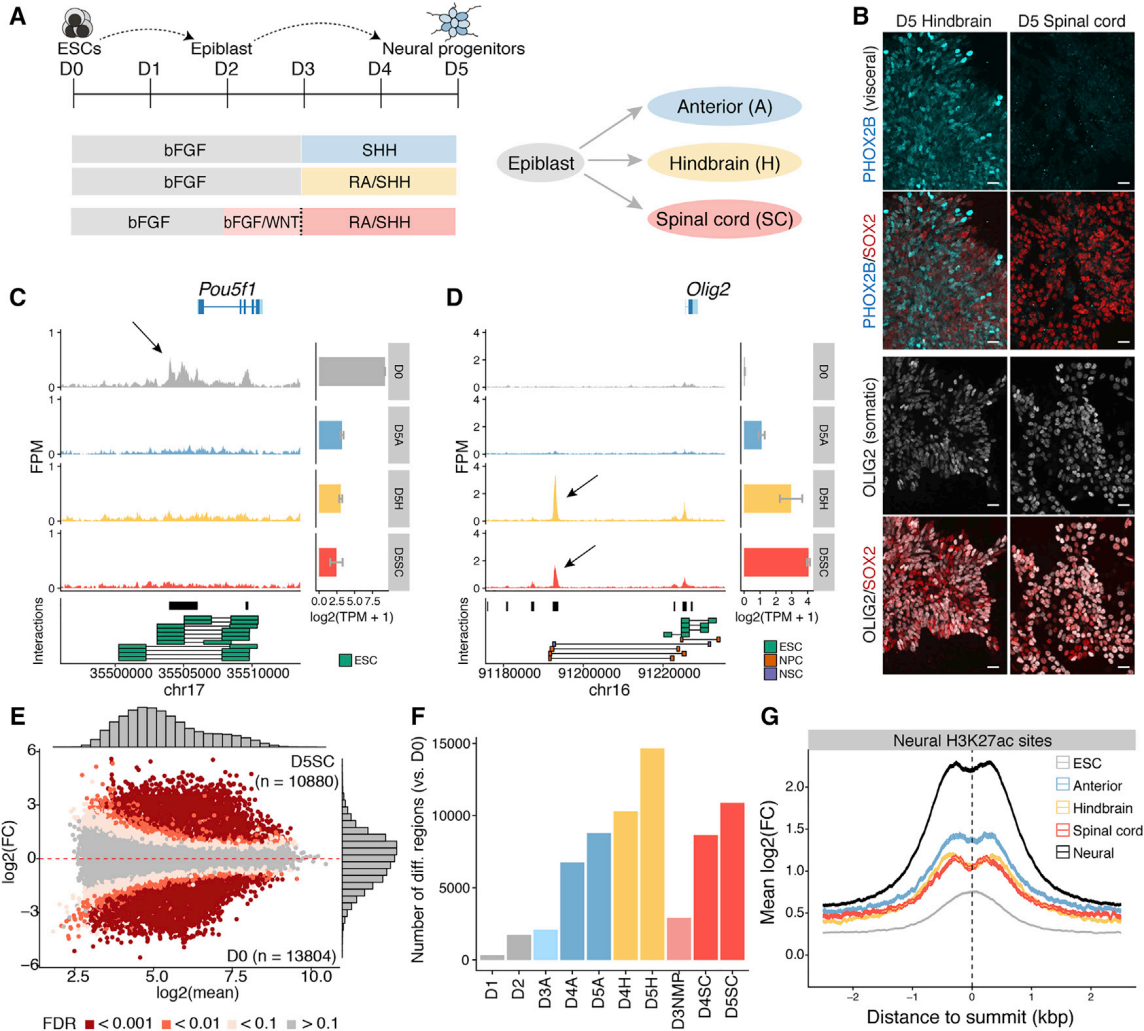
Regulatory Element Usage Distinguishes Cell State during Neural Induction (A) Schematic of mouse ESCs differentiated to NPs with anterior (top), hindbrain (middle), or spinal cord (bottom) identity. Spinal cord progenitors are generated via an NMP state induced by the addition of FGF and WNT signals from day (D) 2 to 3 (light pink shading). (B) D5 immunofluorescence reveals hindbrain progenitors generate a mixture of PHOX2B expressing visceral and OLIG2 expressing somatic MNs. Spinal cord progenitors lack visceral but generate OLIG2 expressing somatic MN progenitors. Scale bars represent 20 μm. (C and D) ATAC-seq accessible regions present in ESCs (D0, gray) compared with D5 anterior (D5A; blue), hindbrain (D5H; yellow), and spinal cord (D5SC; red) progenitors and associated gene expression levels determined by mRNA-seq (Gouti et al., 2014; error bars = SEM). *cis* interactions indicated below each plot represent known genomic interactions from published data (Table S2). ESCs express *Oct4* and show accessibility at *Pou5f1/Oct4* enhancers (C, arrow). D5H and D5SC NPs have open regions flanking neural expressed *Olig2* (D, arrow). (E) Genome-wide accessibility comparison in D5 spinal cord (D5SC) versus D0 ESCs (false discovery rate [FDR] < 0.01 and |log2(FC)| > 1). (F) The proportion of differential sites present in each condition compared with ESCs. (G) Both neural and AP-specific sites, but not ESC sites, are enriched in H3K27ac marks from NPs (Peterson et al., 2012). bFGF, basic fibroblast growth factor; D, day; ESC, embryonic stem cell; FC, fold change; kbp, kilobase pairs; RA, retinoic acid; SHH, sonic hedgehog; TPM, transcripts per million.

To define the sequence of events that commit neural cells to different AP identities, we took advantage of mouse ESCs, which can be differentiated into NPs with anterior (forebrain and/or midbrain), hindbrain, or spinal cord identities (Gouti et al., 2014, Gouti et al., 2017; Figure 1A). By day (D) 5, hindbrain NPs produced visceral motor neuron progenitors expressing PHOX2B and somatic motor neuron progenitors (pMNs) expressing OLIG2, akin to the brainstem (Figure 1B; Gouti et al., 2014, Pattyn et al., 1997). By contrast, spinal cord NPs generated OLIG2-expressing somatic pMNs (Figure 1B) that expressed *Hox* genes characteristic of cervical and thoracic regions (Figures 3G and 3H) but no visceral motor neurons (Figure 1B).

### Chromatin Accessibility Defines NP Identity

**Figure S1:**
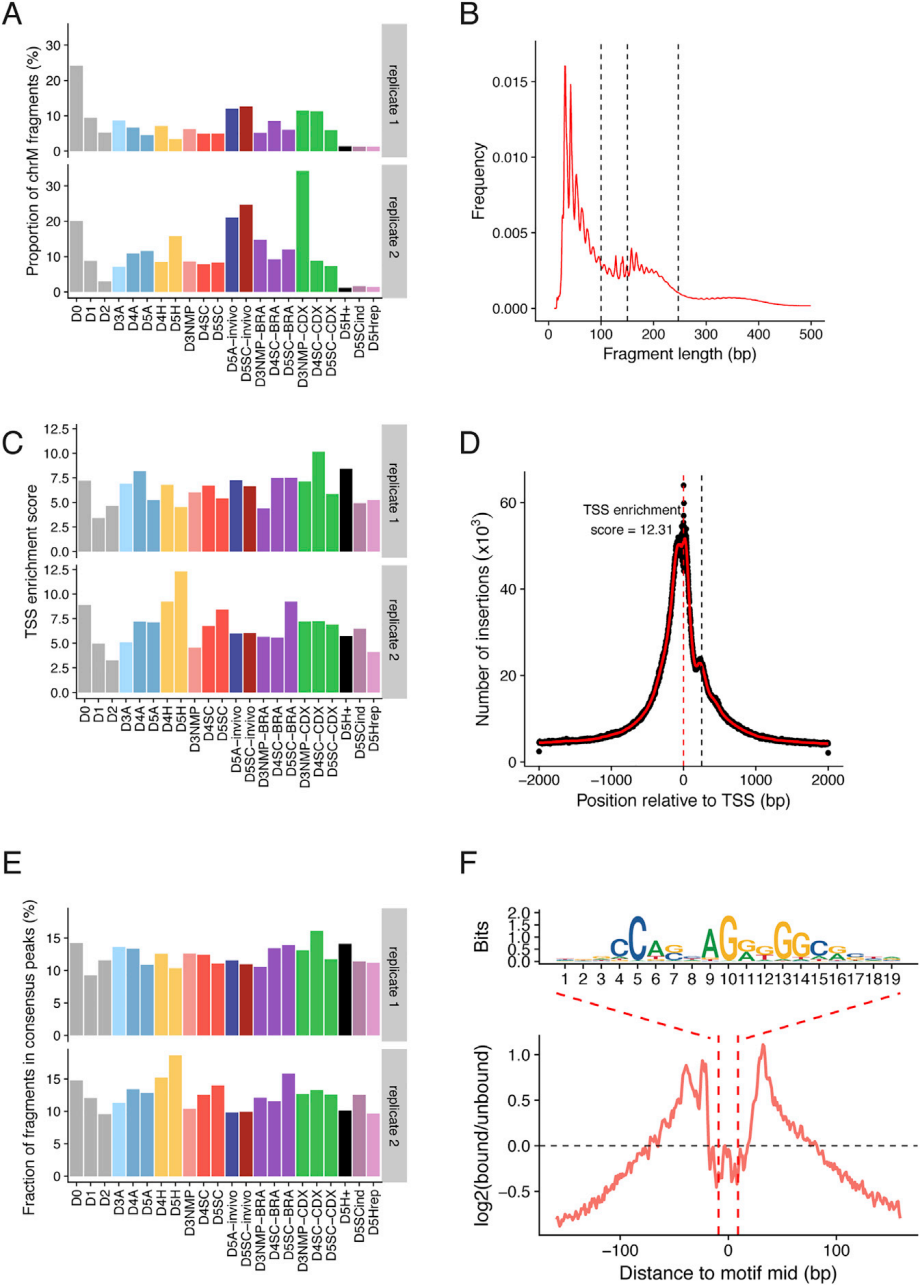
Quality Control for All ATAC-Seq Samples Generated in This Study, Related to STAR Methods and Figure 1 (A) The proportion of mitochondrial fragments recovered across each sample. (B) Representative example showing the distribution of fragment lengths recovered from ATAC-seq, using paired-end sequencing. (C) Average level of Tn5 enrichment (score = maximum(number of insertions)/minimum(number of insertions)) observed across transcription start sites (TSS) for each sample. (D) Summarized Tn5 insertion profile covering ± 2kb around annotated TSS for sample D5H (replicate 2). Red line corresponds to a 50bp running average. (E) The fractions of fragments that map to *in vitro* consensus peak regions. (F) CTCF footprint present in ESC accessible regions as determined by ATAC-seq.

We used ATAC sequencing (ATAC-seq) (Buenrostro et al., 2013) to examine chromatin accessibility in differentiating ESCs (Figures 1C, 1D, and S1). Distinct chromatin accessibility profiles were evident in different cell types; enhancers directing pluripotency genes were accessible in ESCs, but not in any of the three neural conditions (Figure 1C). By contrast, neural enhancers, such as *Olig2* (Oosterveen et al., 2012, Peterson et al., 2012), exhibited the opposite behavior (Figure 1D). Genome-wide comparisons between ESCs and NPs revealed widespread differences in accessibility between D0 ESCs and each of the D5 NPs (Figure 1E). As cells differentiated to NPs, differences in chromatin accessibility progressively emerged (Figure 1F) and sites open in NPs (D5A, D5H, and D5SC) were marked by H3K27ac (Peterson et al., 2012; Figure 1G). Thus, ATAC-seq allows the identification of regulatory regions that define the NP lineage.

### Chromatin Accessibility Differences Define NP Axial Identity

**Figure 2:**
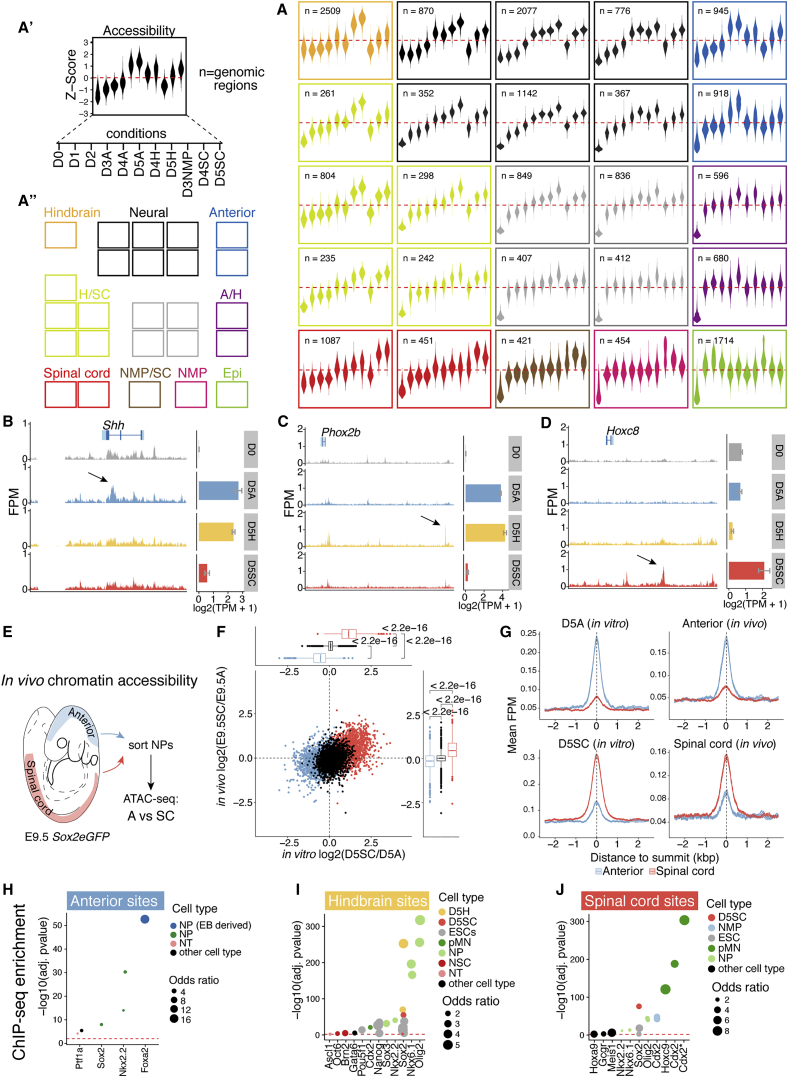
Differential Enhancer Usage Reveals NP Axial Identity (A) Self-organizing map (SOM) of regulatory regions showing differential accessibility relative to D0. Each plot represents the chromatin accessibility *Z* score for each genomic region in the cluster for each condition (see key in A’). Many sites are common (“neural sites”) to all NPs (black cluster; n = 5,584). These differ from epiblast regulatory regions that are accessible at early stages of the differentiation (Epi; green; n = 1,714). Region-specific sites are identified in anterior (blue; n = 1,863), hindbrain (orange; n = 2,509), and spinal cord (red; n = 1,538) progenitors. A distinct set of regulatory regions identifies D3 NMPs (pink; n = 454 regions). A/H represents shared anterior and hindbrain accessible sites (purple; n = 1,276); H/SC, shared hindbrain and spinal cord sites (lime; n = 1,840); and NMP/SC, shared NMP and spinal cord sites (brown; n = 421). Grey are unclassified sites. (B–D) Examples of ATAC-seq accessible regions that define anterior (B), hindbrain (C), or spinal cord (D) D5 progenitors, identified using the SOM (A). Gene expression from mRNA-seq (error bars = SEM) is shown to the right. Anterior progenitors display region-specific open sites at *Shh* (B), and hindbrain progenitors demonstrate a *Phox2b* site (C) and a *Hoxc8* site opens in spinal cord (D). (E–G) *In vivo* ATAC-seq correlates with *in vitro*. NPs obtained from brain (E; blue shading) and spinal cord (E; red shading) of E9.5 *Sox2eGFP* embryos. The fold change in accessibility at anterior (blue; n = 1,863) and spinal cord (red; n = 1,538) sites identified *in vitro* in spinal cord NPs relative to anterior NPs *in vivo* correlates with AP identity (F). Common neural sites *in vitro* (black) are similar in both populations *in vivo*. Anterior sites identified *in vitro* show preferential accessibility *in vivo* in anterior NPs (G), and spinal cord *in vitro* sites show more accessibility *in vivo* in spinal cord NPs (p values; Wilcoxon rank-sum test). (H–J) ChIP-seq enrichment analysis in anterior (H), hindbrain (I), and spinal cord sites (J). SOX2 ChIP-seq in D5 hindbrain (D5H) and spinal cord (D5SC) cells reveals that the binding site preference is condition specific (I and J). CDX2^∗^ denotes CDX2 ChIP-seq performed in the presence of FGF signaling (Mazzoni et al., 2013). FPM, fragments per million; neural EB, embryoid bodied-derived NPs; NMP, neuromesodermal progenitors; NP, neural progenitors; NT, neural tube; pMN, motor neuron progenitors. See also Figure S2.

**Figure S2:**
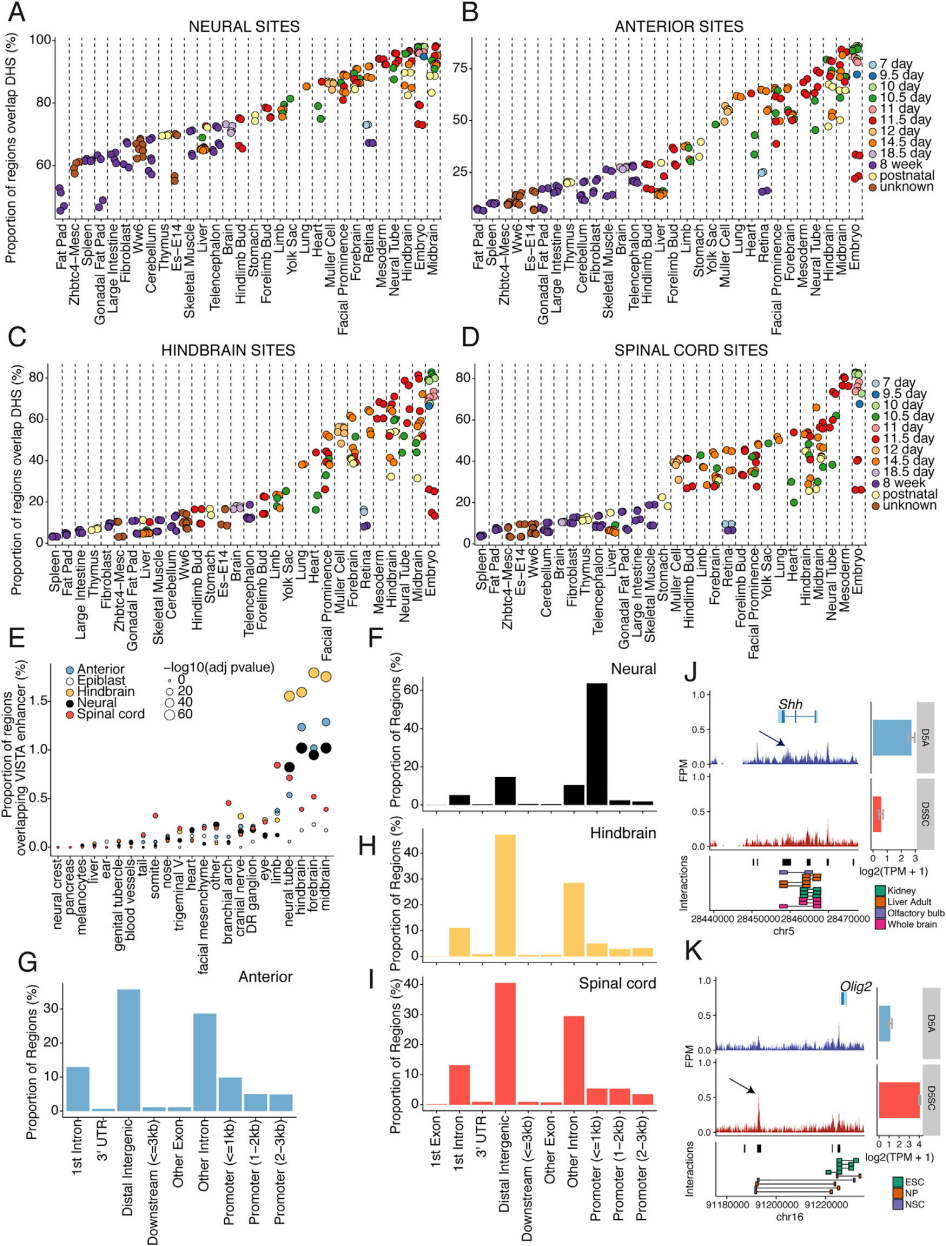
Tissue Specificity and Genomic Location of Regulatory Regions that Define Neural and Region-Specific Identity, Related to Figure 2 (A–D) Comparison of ATAC-seq identified regions with previously published DNase hypersensitivity sites present across a range of *in vivo* tissues and time points from the ENCODE regulatory element database (ENCODE Project Consortium, 2012, Sloan et al., 2016). Genomic regions correspond to neural (A), anterior (B), hindbrain (C) and spinal cord (D) specific sites from Figure 2A. Each set of genomic regions demonstrates an enrichment in embryonic and neural samples *in vivo*. (E) Comparison of ATAC-seq identified regions with the Vista enhancer database (Visel et al., 2007) shows that accessible regions correspond to enhancers that have neural tissue specificity *in vivo*. (F–I) Classification of neural (F), anterior (G), hindbrain (H) and spinal cord (I) sites according to genomic position. Neural sites are enriched at promoter regions (F), in contrast to the region-specific sites, which predominantly occupy distal intergenic and intronic regions (G-I). p values determined using one-sided Binomial test and multiple testing corrected using Benjamini–Hochberg procedure. (J and K) *In vivo*-derived neural progenitors display accessibility at known enhancers depending on their axial identity. Genome browser view (mm10 assembly) showing ATAC-seq from anterior (blue track) and spinal cord (red track) neural progenitors obtained from E9.5 mouse embryos at the *Shh* (J) and *Olig2* (K) locus. Arrows indicate known enhancers that direct *Shh* expression in the midbrain (Epstein et al., 1999; J) and *Olig2* in the spinal cord (Oosterveen et al., 2012 and Peterson et al., 2012; K). Gene expression levels determined by mRNA-seq (Gouti et al., 2014) are shown as bar plots from *in vitro* D5 anterior (blue) and spinal cord (red) conditions (error bars = SEM). Chromatin interactions recovered from indicated tissues are presented below for comparison. Peak regions are represented with black bars. A = anterior neural progenitor; DR = dorsal root; NP = neural progenitor; NSC = neural stem cell; SC = spinal cord progenitor; TPM = transcripts per million.

Unsupervised clustering using self-organizing maps (SOMs) (Törönen et al., 1999) of regulatory regions that changed accessibility after removal of ESCs from pluripotent conditions produced 10 distinct groups, each with their own accessibility dynamics (Figures 2A–2A”). A large number of regions (n = 5,584) were accessible in all NP conditions. These overlap with DNaseI-hypersensitive sites (DHSs) that are accessible in neural tissues (Figure S2A; ENCODE Project Consortium, 2012, Sloan et al., 2016). We refer to these sites as “neural sites.”

In addition, sets of regulatory regions became accessible in NPs depending on their AP identity: 1,863 sites were enriched in anterior NPs (Figure 2A, blue cluster); 2,509 in hindbrain progenitors (Figure 2A, orange cluster); and 1,538 in spinal cord progenitors (Figure 2A, red clusters). Examining the position of these “region-specific” regulatory sites indicated that these also overlapped with open chromatin sites and displayed activity in neural tissues (Visel et al., 2007; Figures S2B–S2E). In contrast to common neural sites, mainly (∼63.8%) located close to the transcriptional start site (TSS) of coding genes, region-specific sites were predominantly in distal regions of the genome (Figures S2F–S2I).

Gene-to-peak associations indicated that region-specific sites flanked many neural genes and reflected AP identity (Figures 2B–2D; Table S1). Anterior NPs displayed region-specific sites at *Shh* (Figure 2B), overlapping the previously identified *Shh* brain enhancer (Epstein et al., 1999). By contrast, hindbrain region-specific sites flanked the cranial MN gene *Phox2b* (Figure 2C), and in the spinal cord, region-specific sites flanked many posterior *Hox* genes, including *Hoxc8* (Figure 2D), in addition to neural genes, such as *Nkx6-1*, *Lhx1*, *Sox11*, and *Sox2* (Table S1).

To test whether the region-specific signatures observed *in vitro* reflect *in vivo* differences, we performed ATAC-seq on mouse NPs isolated from different AP levels of embryonic day 9.5 (E9.5) embryos (Figures 2E–2G). Neural sites common to all *in vitro* NPs were equally enriched in both populations *in vivo* (Figure 2F). By contrast, anterior *in vivo* NPs demonstrated increased accessibility at sites that define *in vitro* anterior neural identity (Figures 2G and S2J). Similar results were obtained when examining *in vivo* derived spinal cord NPs: increased accessibility was observed in regions that define spinal cord NPs *in vitro* (Figures 2G and S2K). In summary, chromatin accessibility changes *in vitro* predict AP position *in vivo*.

### Context-Dependent Binding of Neural TFs Defines Axial Identity

**Figure S3:**
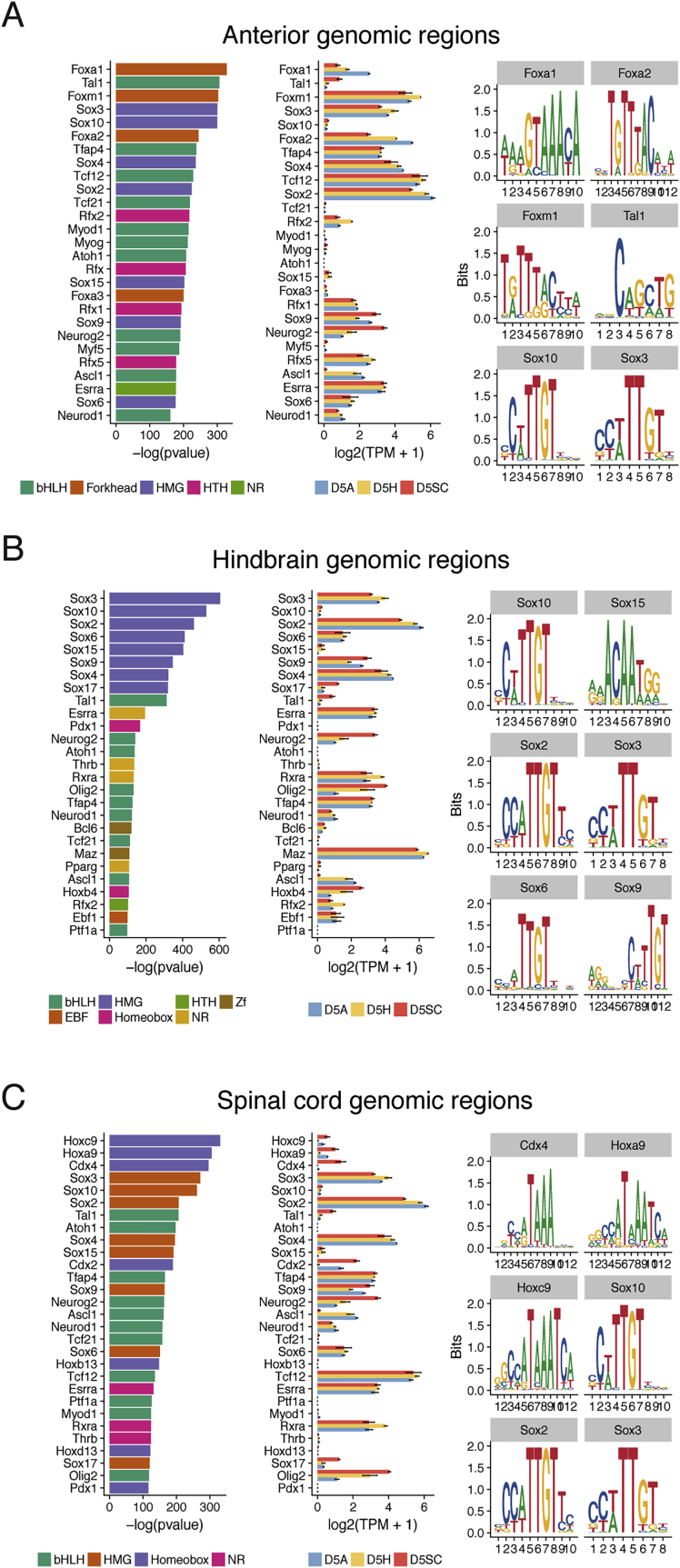
Motif Analysis of Region-Specific Sites that Define Anterior, Hindbrain, and Spinal Cord, Related to Figure 2 (A–C) Motif enrichment analysis performed using Homer on anterior (A), hindbrain (B) and spinal cord (C) specific sites shows distinct and common neural factors enriched at each AP level. For each factor (indicated on the left), the corresponding expression level determined by mRNA-seq (Gouti et al., 2014) in the same condition at D5 of the *in vitro* differentiation is shown (central column; error bars = SEM). The top 6 predicted motif logos are presented on the right. TPM = transcripts per million.

We performed chromatin immunoprecipitation (ChIP)-seq enrichment analysis using a set of publicly available datasets (Sheffield and Bock, 2016; Table S2). This revealed FOXA2 and NKX2-2 binding sites in anterior (Figure 2H), OLIG2 and NKX6-1 in the hindbrain (Figure 2I), and CDX2 and HOXC9 in spinal cord NPs (Figure 2J). Motif enrichment predicted SoxB1 TF motifs (SOX1/2/3) in all three NPs (Figures S3A–S3C), consistent with their expression throughout the neuraxis (Kamachi and Kondoh, 2013, Wood and Episkopou, 1999). Notably, hindbrain and spinal cord cells, which are both exposed to the same signals (retinoic acid [RA]/sonic hedgehog [SHH]) from D3 to D5, are enriched for SOXB1 binding but at distinct genomic sites (Figures S3B and S3C). The presence of posterior HOX binding together with SOX in spinal cord progenitors suggested that region-specific expression of *Hox* genes influenced the binding site preference of the core neural SOXB1 TFs (Hagey et al., 2016).

To validate the *in silico* results, we performed ChIP-seq of the SOXB1 TF SOX2 in D5 hindbrain and spinal cord NPs (Figures 2I and 2J). This confirmed hindbrain-predicted SOX sites were engaged with SOX2 in the hindbrain, but not spinal, NPs. Conversely, SOX2-accessible sites specific to the spinal cord showed increased engagement of SOX2 in spinal cord versus hindbrain conditions (Figures 2I and 2J). These data indicate that NPs develop region-specific transcription factor binding patterns.

### Developmental Timing Determines Posteriorization

**Figure 3:**
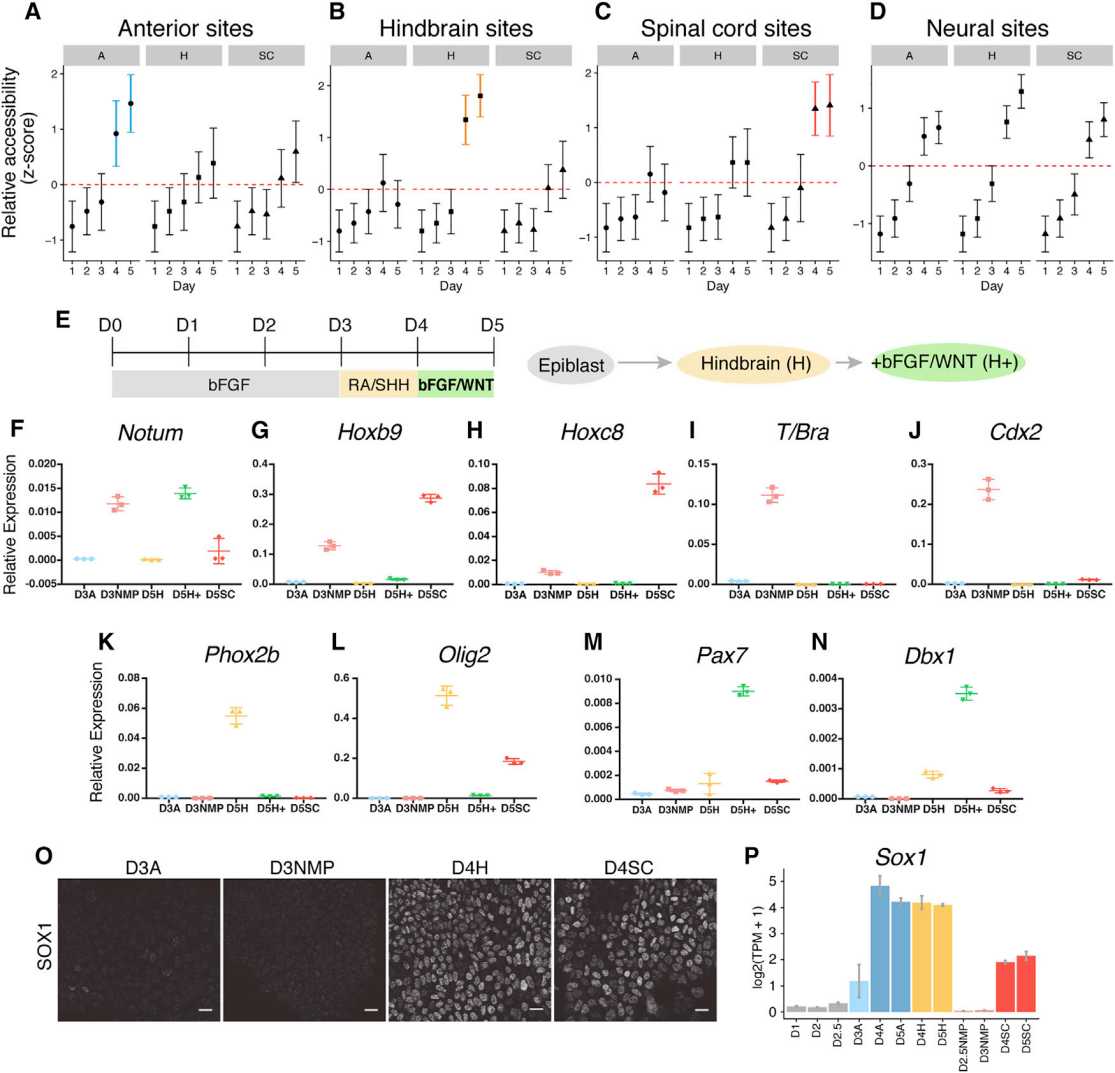
Axial Identity Is Established in Cells prior to Neural Identity (A–C) The average accessibility (*Z* score) of region-specific sites over time in anterior (labeled “A”), hindbrain (labeled “H”), or spinal cord (labeled “SC”) conditions. AP-specific sites become accessible between D3 and D4. Spinal cord progenitors do not transiently open sites corresponding to anterior (A) or hindbrain (B) identity before opening spinal cord-specific sites (C). (D) Neural sites become accessible in all regions at the same time. Error bars = SD. (E) Schematic of the differentiation (H+ condition). (F–N) qRT-PCR of genes at D3 and D5 following the differentiation of cells to hindbrain (D5H), spinal cord (D5SC), or “hindbrain+” (D5H+) identity. The WNT target *Notum* (F) is observed following WNT signaling treatment at D3 (D3NMP) and D5 (D5H+). Induction of posterior spinal cord *Hox* genes *Hoxb9* and *Hoxc8* is dependent on timing: induction in D3NMP follows D2 to D3 treatment with WNT signals, but not at D5 in D5H+ cells following D4 to D5 treatment with the same signals (G and H). Induction of *T/Bra* and *Cdx2* is dependent on timing, responding to early (D2 to D3), but not late (D4 to D5), treatment with WNT signals (I and J). Late treatment of WNT in the D5H+ condition prevents expression of ventral neural genes *Phox2b* and *Olig2* (K and L, compare D5H to D5H+) while dorsal *Pax7* (M) and intermediate *Dbx1* (N) neural tube genes are induced. Error bars represent the standard deviation. (O) SOX1 immunofluorescence on D3 versus D4 cells cultured in hindbrain (D3A and D4H) or spinal cord (D3NMP and D4SC) conditions. Scale bars represent 20 μm. (P) *Sox1* expression, detected by mRNA-seq (Gouti et al., 2014), at the indicated times and conditions. Error bars = SEM.

To generate spinal cord, the prevailing view is that anterior neural cells are gradually posteriorized (Stern, 2001). However, by examining region-specific sites over time, we found that spinal cord cells failed to exhibit transient accessibility at either anterior or hindbrain sites (Figures 3A and 3B). Instead, spinal cord-specific sites became accessible in spinal cord conditions by D4 of the differentiation (Figure 3C). This coincides with accessibility at neural sites (Figures 3A–3D).

**Figure S4:**
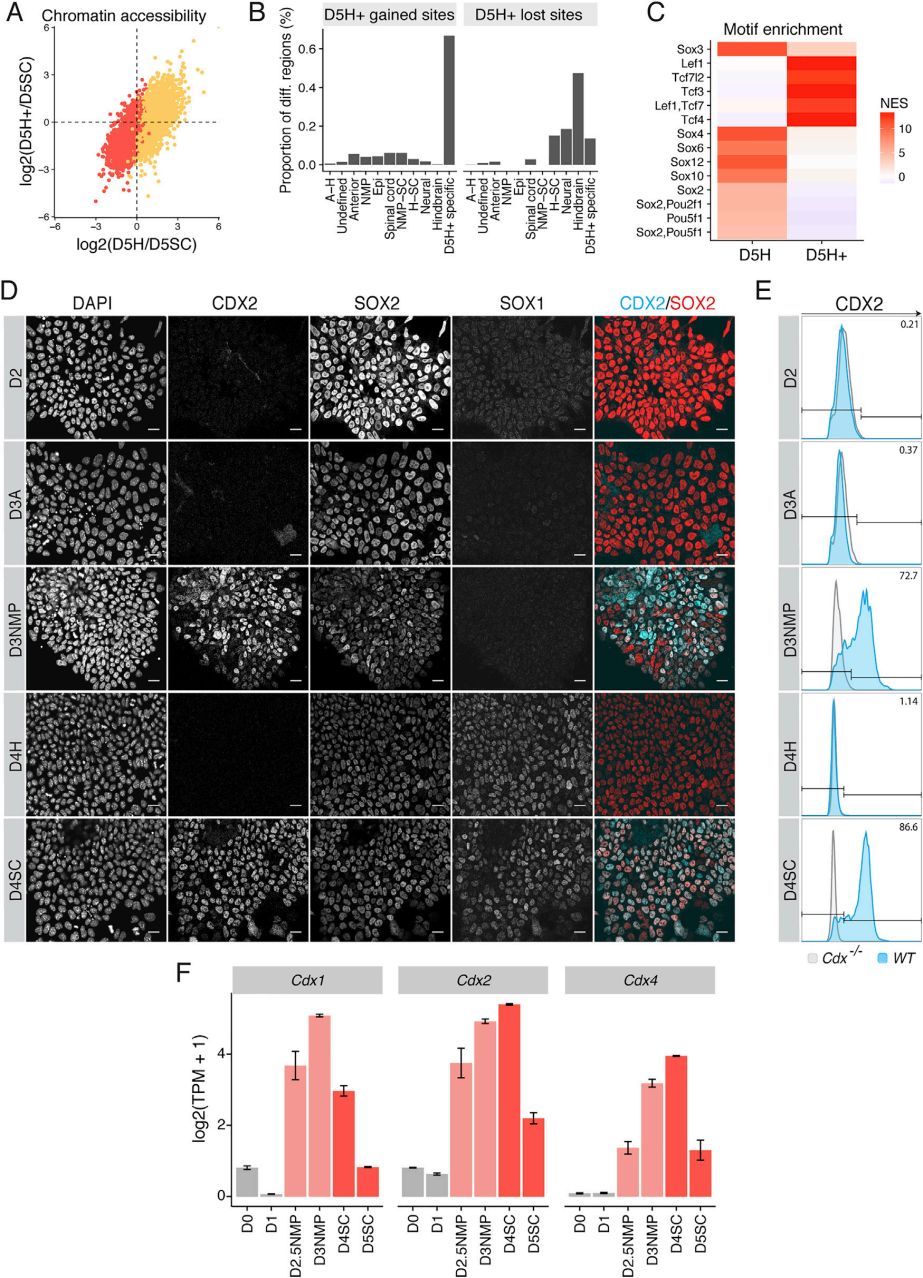
Expression Dynamics of *Cdx* TFs during the Spinal Cord Differentiation, Related to Figure 4 (A) Scatterplot showing the fold change of D5H+ or D5H cells relative to the D5SC condition. Note that the majority of hindbrain sites (yellow) remain accessible in D5H+ treated cells, in contrast to spinal cord (red) sites. (B) The distribution of genomic regions, as defined in the self-organizing map (SOM; see Figure 2A), in D5H+ cells that show changes in accessibility when compared to D5H cells (FDR < 0.01 & |log2(FC)| > 1). Note that D5H+ cells do not gain sites associated with D3NMP, which are treated with WNT between D2-3 (see columns labeled “NMP”), but gain additional sites not classified in the SOM (see columns labeled “D5H+ specific). (C) Motif enrichment using iCis Target (Imrichová et al., 2015) on genomic regions that are differentially accessible between D5H and D5H+ reveals an enrichment of TCF/Lef factors following WNT treatment in D5H+ cells, in contrast to the D5H condition which harbours sites enriched in SOX factors. (D) Confocal microscopy of hindbrain and spinal cord cells from D2-4 shows the induction of CDX2 at D3 in FGF/WNT in the spinal cord condition (D3NMP). SOX1 expression, marking neural progenitors, is not detected until D4 in both conditions, following RA and SHH treatment. Right column shows CDX2/SOX2 composites. (E) Flow cytometry in WT (blue) versus *Cdx* triple mutant ESCs (grey) at the indicated time points and conditions indicates the percentage of SOX2 positive cells that express CDX2. (F) Expression profile determined by mRNA-seq (Gouti et al., 2014) for *Cdx1*, *Cdx2* and *Cdx4* from D0 to D5 of the spinal cord differentiation. Error bars = SEM. NMP=neuromesodermal progenitor; SC=spinal cord; TPM=transcripts per million.

Although hindbrain and spinal cord NPs are exposed to the same (RA/SHH) signals, they adopt region-specific signatures (Figures 3B and 3C). Unlike the hindbrain, *in vitro* generation of spinal cord NPs requires exposure to FGF/WNT signals from D2 to D3 (Figures 1A and 3E). Provision of a 24-hr pulse of FGF/WNT signals between D4 and D5 under hindbrain conditions resulted in the induction of the canonical WNT signaling target *Notum* (Figure 3F, compare D3NMP and D5H+). However, D4 to D5 treatment was not sufficient to induce expression of *Hox* genes characteristic of the spinal cord (Figures 3G and 3H). Likewise, the induction of *Brachyury* (*T/Bra*) and *Cdx2*, normally induced at D3 by a D2 to D3 pulse of FGF/WNT (Figures 3I, 3J, and S4F), was not observed at D5 following FGF/WNT treatment between D4 and D5 (Figures 3I and 3J).

ATAC-seq on D5H+ cells revealed that hindbrain sites remained accessible (Figures S4A and S4B). The few changes in accessibility found in D5H+ cells showed an enrichment in TCF/LEF motifs, indicative of WNT signaling (Figures S4B and S4C). D5H+ cells failed to express ventral hindbrain markers *Phox2b* and *Olig2* (Figures 3K and 3L) and instead expressed dorsal (*Pax7*) and intermediate (*Dbx1*) neural tube genes (Figures 3M and 3N). Thus, prior to NP formation (D2 to D3), FGF/WNT signals exert posteriorizing effects; after neural induction (D4 to D5), the same extrinsic signals promote dorsal identity (Figures 3O and 3P; Muroyama et al., 2002).

### WNT Drives Transient Chromatin Accessibility

**Figure 4:**
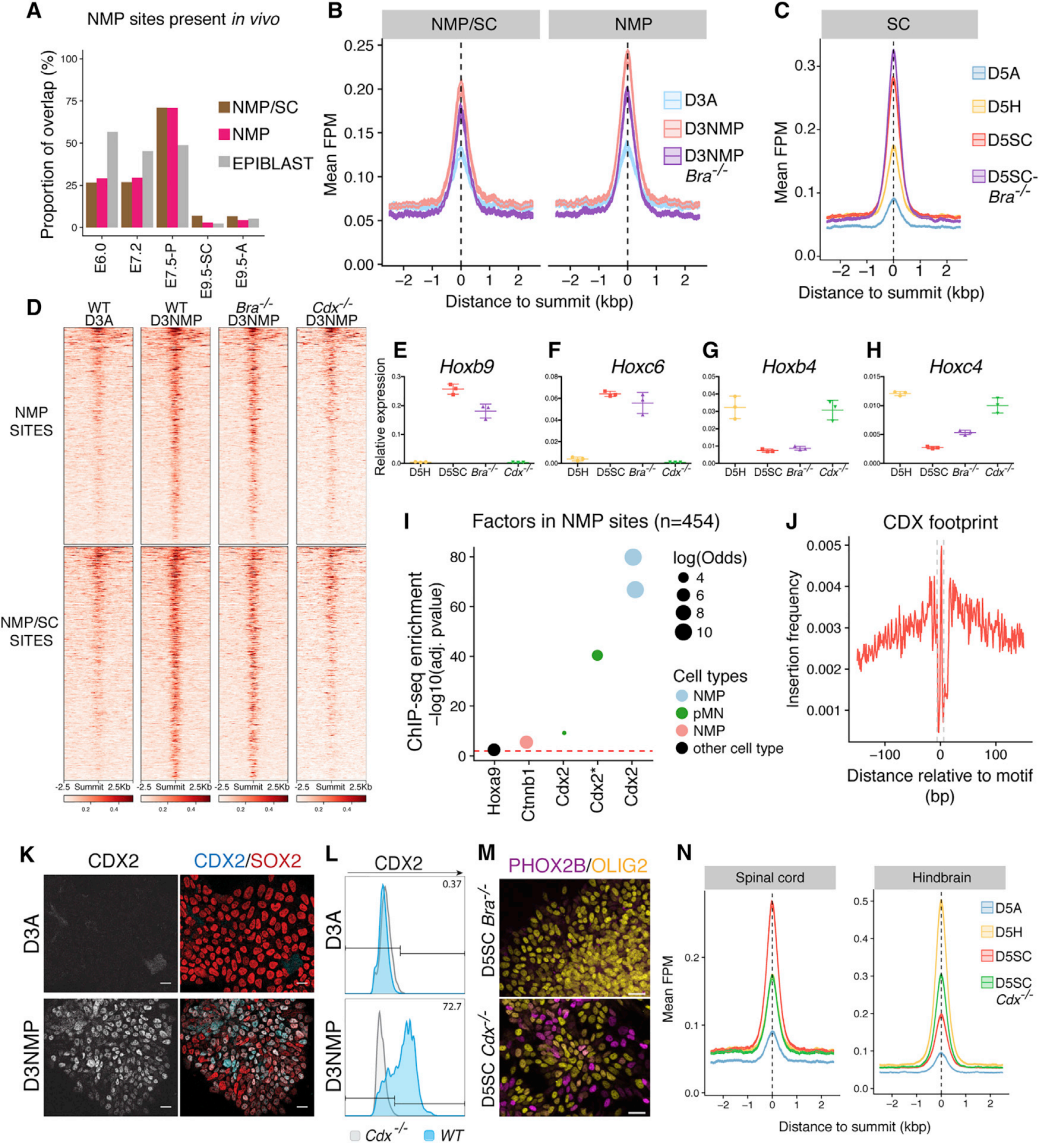
WNT Establishes Spinal Cord Identity via CDX-Dependent Chromatin Remodeling (A) Proportion of NMP, NMP/SC, or epiblast genomic sites from the SOM (Figure 2A) that overlap with accessible regions *in vivo* (Neijts et al., 2016; this study). 71% of all NMP sites identified *in vitro* are found in the posterior E7.5 embryo (E7.5-P; Neijts et al., 2016). This contrasts with NPs from the spinal cord (E9.5-SC) or anterior nervous system (E9.5-A; this study), which show little overlap with these sites. (B) The average accessibility profile of NMP/SC and NMP-specific sites in wild-type versus *T/Bra*^*−/−*^ mutant cells. These sites remain accessible in *T/Bra*^*−/−*^ mutant cells at D3 of the spinal cord differentiation. (C) *T/Bra*^*−/−*^ mutant cells differentiated to D5 under spinal cord conditions retain accessibility at spinal cord genomic sites. (D) Heatmap showing NMP (top panels) and NMP/SC (bottom panels) site accessibility in D3NMP conditions from WT, *T/Bra*^*−/−*^, and *Cdx1/2/4* (*Cdx*^*−/−*^) mutant cells. These sites are maintained in the absence of *T/Bra* but are reduced in the absence of the three *Cdx* TFs. (E–H) qRT-PCR of *Hox* genes at D5 indicates AP identity of hindbrain (D5H) and spinal cord (D5SC) cells in wild-type compared with *T/Bra*^*−/−*^ and *Cdx*^*−/−*^ mutant cells differentiated under spinal cord conditions. *Bra*^*−/−*^ mutant cells retain expression of spinal cord *Hox* genes *Hoxb9* (E) and *Hoxc6* (F) in contrast to *Cdx* mutant cells, which express *Hoxb4* (G) and *Hoxc4* (H). Error bars represent the standard deviation. (I) ChIP-seq enrichment analysis reveals that CDX2 is highly enriched at NMP-specific sites (p values; one-sided Fisher’s exact test; multiple testing corrected using Benjamini-Hochberg procedure). (J) Tn5 insertion frequency, across all SOM regions containing at least one CDX2 motif, at nucleotide resolution in D3NMP cells reveals the presence of a footprint centered on the CDX2 motif. (K) CDX2 (cyan) and Sox2 (red) immunofluorescence at D3. Scale bars represent 20 μm. (L) Histogram of CDX2-positive cells at D3 comparing WT (blue) with *Cdx*^*−/−*^ mutant cells (gray), determined by flow cytometry. Numbers indicate percentage of SOX2-positive cells that are CDX2 positive. (M) Removal of the three *Cdx* transcription factors *Cdx1*/2/4 results in the continued induction of OLIG2 (gold) and ectopic production of cranial MN progenitors, marked by PHOX2B (magenta). Scale bars represent 20 μm. (N) The average profile of spinal cord sites (left plot) shows that, relative to D5 spinal cord (D5SC, red), accessibility at these sites is reduced in *Cdx*^*−/−*^ mutant cells differentiated under the same conditions (D5SC *Cdx*^*−/−*^, green), to the same extent as D5 hindbrain cells (yellow). Under spinal cord conditions, *Cdx* mutant cells show increased accessibility at hindbrain sites (right plot).

To understand how FGF/WNT signals exert a stage-specific posteriorizing effect on cells, we examined chromatin accessibility in cells at D3 following WNT treatment. We found accessibility at 875 unique regions (Figure 2A; NMP/SC cluster n = 421 regions; NMP cluster n = 454 regions) that fail to appear in D5H+ cells that received FGF/WNT treatment from D4 to D5 (Figure S4B). Of these 875 sites, 454 (51.8%) were immediately downregulated as cells committed to spinal cord fates (Figure 2A, NMP cluster). We asked to what extent these sites overlap with chromatin accessibility *in vivo*. We took advantage of ATAC-seq data collected from mouse epiblasts at E6.0–E7.2 and from E7.5 posterior mouse tissue (Neijts et al., 2016). We found more than 71% of sites induced by FGF/WNT signaling *in vitro* at D3 overlapped with accessible sites found in E7.5 posterior mouse embryos, although the overlap with either the epiblast (29% at E6.0) or purified NPs (<5% at E9.5) is much less (Figure 4A). We also found that the epiblast-specific sites defined in the SOM (Figure 2A, epiblast cluster) showed the greatest overlap with those found *in vivo* in E6.0 epiblast. Very little overlap was evident with *in vivo* NPs (Figure 4A). These data suggest that the chromatin accessibility signatures identified *in vitro* correspond to their respective, tissue-specific, regulatory signatures *in vivo*.

These data indicate that, as cells transition to a spinal cord identity, they transiently adopt a genomic signature distinct from both epiblast and neural tissue. This transition state includes the bipotential population of NMPs, which contribute to both the spinal cord and somites (Gouti et al., 2014, Tsakiridis et al., 2014, Turner et al., 2014, Tzouanacou et al., 2009). It remained possible that the NMP-accessible sites represented nascent mesoderm. To test this, we used ESCs lacking *T/Bra* that form spinal cord NPs, but not paraxial mesoderm (Gouti et al., 2014). Similar to wild-type (WT) cells, WNT treatment continued to drive accessibility at NMP (r = 0.93) and NMP/SC (r = 0.95) sites in the absence of *T/Bra* (Figures 4B and 4D). Furthermore, *T/Bra*-lacking ESCs differentiated to D5SC maintained spinal cord sites (Figure 4C) and the expression of posterior *Hox* genes, *Hoxb9* and *Hoxc6* (Figures 4E and 4F), and expression of *3′ Hox* genes (*Hoxb4* and *Hoxc4*) was reduced, similar to WT D5SC cells (Figures 4G and 4H). Thus, T/BRA is dispensable during the chromatin remodeling events driven by WNT signaling at NMP and NMP/spinal cord sites (Figures 4B and 4D).

### The Acquisition of Spinal Cord Fate Requires CDX to Repress Hindbrain Identity

To establish which factors mediate WNT-dependent chromatin remodeling, we performed ChIP-seq enrichment analysis on NMP sites and identified an enrichment in CDX2 (Figure 4I). Nucleotide resolution analysis verified the presence of a CDX “footprint” in D3NMP cells (Figure 4J), suggesting CDX occupancy at these sites. At the single-cell level, CDX2 protein is detected in the majority of D3 cells following FGF/WNT (Figures 4K, 4L, and S4D), preceding expression of NP markers, such as SOX1 (Figures 3O, 3P, and S4D).

WNT signaling is critical for elongation of the trunk *in vivo* (Greco et al., 1996, Nowotschin et al., 2012, Olivera-Martinez et al., 2012), and genetic removal of *Cdx* TFs or combined absence of *Cdx2* and *T/Bra* results in severe axis elongation defects (Amin et al., 2016, van Rooijen et al., 2012, Young et al., 2009). We took advantage of the *in vitro* system to uncouple cell fate decisions from axis elongation. To test whether CDX TFs are necessary for the generation of spinal cord cells, we examined chromatin accessibility in cells lacking all three CDX factors, *Cdx1*, *2*, and *4* (*Cdx*^*1,2,4−/−*^) (Gouti et al., 2017). In contrast to the loss of *T/Bra*, the elimination of all three *Cdx* genes had a profound effect on the response to WNT signaling (Figure 4D). Both NMP and NMP/SC shared sites were reduced, with accessibility levels correlating more with D3A cells (NMP sites r = 0.87; NMP/SC sites r = 0.88) than to D3NMP cells (NMP sites r = 0.65; NMP/SC sites r = 0.78). This suggests that CDX factors are essential for the remodeling of chromatin accessibility associated with an NMP state (Amin et al., 2016) as well as the transition from an NMP to spinal cord fate (Figure 4D). Furthermore, the differentiation of mutant *Cdx* cells to NPs no longer resulted in the expression of spinal cord *5′ Hox* genes *Hoxb9* and *Hoxc6* (Figures 4E and 4F). Instead, *Cdx* mutant cells expressed *Hoxb4* and *Hoxc4* and generated visceral pMNs marked by PHOX2B (Figures 4G, 4H, and 4M). Sustained *Olig2* induction was observed, suggesting that the removal of CDX TFs does not impede NP establishment (Figure 4M). Analysis of the chromatin accessibility in D5 *Cdx*^*1,2,4−/−*^ cells differentiated under spinal cord conditions revealed that these cells also lacked accessibility at spinal cord sites and instead gained hindbrain sites (Figure 4N).

### CDX2 Can Substitute for WNT and Prolong Spinal Cord Competency

**Figure 5:**
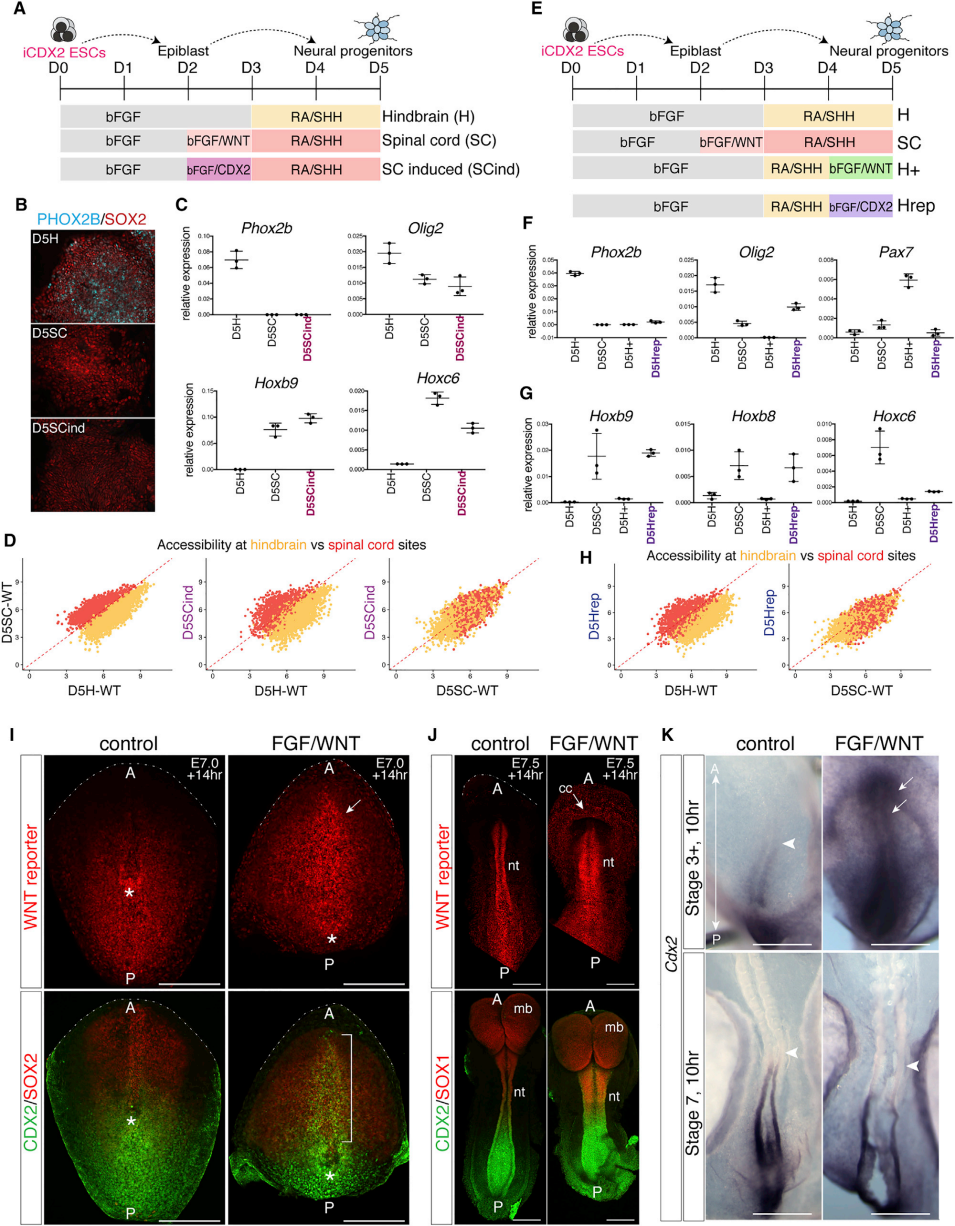
CDX2 Can Replace WNT and Prolong Spinal Cord Competency (A) Schematic of the differentiation using iCDX2 ESCs (Niwa et al., 2005) to induce CDX2 between D2 and D3 (SCind). (B) Immunofluorescence of NPs at D5 PHOX2B (cyan) and SOX2 (red). Cranial MNs in hindbrain, but not spinal cord or SCind. (C) qRT-PCR analysis at D5 shows that the induction of CDX2 between D2 and D3 maintains *Olig2* expression and upregulates *Hoxb9* and *Hoxc6*. Error bars represent the standard deviation. (D) Chromatin accessibility, measured by ATAC-seq, at hindbrain (yellow) and spinal cord (red) sites at D5. Spinal cord sites are more open in WT spinal cord cells than WT hindbrain cells (left plot). The induction of CDX2 between D2 and D3 (D5SCind) increases accessibility at spinal cord sites versus D5H WT cells (middle plot) and similar levels of accessibility in hindbrain and spinal cord sites when compared to D5SC WT cells (right plot). (E) Schematic of the differentiation using iCDX2 ESCs to induce CDX2 between D4 and D5 under hindbrain conditions (Hrep) versus hindbrain+ (H+) conditions, in which WNT signaling is activated between D4 and D5. (F and G) qRT-PCR data indicate that, by D5, the induction of CDX2 is sufficient to repress *Phox2b* but maintain *Olig2* (F), in contrast to H^+^ cells, which repress *Olig2* expression (Figure 3). The induction of CDX2 between D4 and D5 upregulates posterior *Hox* genes *Hoxb9*, *Hoxb8*, and *Hoxc6* (G). Error bars represent the standard deviation. (H) Accessibility at hindbrain (yellow) and spinal cord (red) sites reveals that D5Hrep cells display increased accessibility at spinal cord sites and loss of hindbrain sites compared to D5H cells. (I and J) WNT reporter embryos cultured for 14 hr in control versus WNT signaling conditions (Table S3) from E7.0 (I) or E7.5 (J). Images show embryos cultured in media containing bFGF (control) versus FGF and CHIR99021 (FGF/WNT) conditions. Embryos are oriented with anterior at the top of the image. Dashed white lines demarcate the anterior limit. (I) Ventral view of E7.0 cultured embryos. Ectopic induction of WNT activity (n = 10/13) and CDX2 (green, white bracket) is observed in the presence of WNT signaling (n = 10/13), but not control conditions (n = 0/10). SOX2 marks the epiblast (red). Asterisk demarcates the position of the node. Scale bars represent 250 μm. (J) Ectopic induction of WNT signaling in E7.5 cultured embryos (n = 13/16) versus control conditions (n = 0/6). No CDX2 (green) expansion was detected in the anterior neural plate (n = 0/31) marked by SOX1 (red) versus control conditions (n = 0/30). Top panels in (J) show ventral views; bottom panels in (J) show dorsal views. Scale bars represent 250 μm. (K) Chick whole-mount *in situ* hybridization for *Cdx2* following 10-hr *ex vivo* embryo culture from HH stage 3+ (top panels) and stage 7 (bottom panels). The addition of WNT signals promotes ectopic (white arrows) anterior *Cdx2* expression at early stages (15/15 in the FGF/WNT versus n = 0/13 control at stage 3+). By contrast, no expansion is observed in response to WNT signaling in stage 7 embryos that already contain a neural plate (n = 0/9 in FGF/WNT and n = 0/12 in control). White arrowheads mark the anterior limit of *Cdx2* expression. Scale bars represent 500 μm. cc, cardiac crescent; mb, midbrain; nt, neural tube; p, posterior.

We asked whether CDX activity could substitute for WNT signals. To this end, we used a 4-hydroxytamoxifen-(TAM) inducible CDX2 ESC line (iCDX2 ESCs) (Niwa et al., 2005). iCDX2 ESCs differentiated under hindbrain or spinal cord conditions (Figure 5A) behaved similarly to WT cells, generating PHOX2B pMNs in the hindbrain condition (Figures 5B and 5C). By inducing CDX2 between D2 and D3, in the absence of WNT signals, PHOX2B was no longer observed at D5, whereas spinal cord *Hox* genes (*Hoxb9* and *Hoxc6*) were expressed (Figures 5B and 5C). We refer to these cells as “spinal cord induced” (SCind). ATAC-seq of D5SCind cells revealed accessibility at spinal cord sites, whereas hindbrain sites were less accessible (Figure 5D). Thus, induction of CDX2 prior to the acquisition of neural identity is sufficient to establish a chromatin accessibility signature typical of spinal cord cells.

We next tested whether CDX2 could promote spinal cord identity in hindbrain progenitors, a cellular context in which FGF/WNT signals had lost this ability (Figure 3D). iCDX2 cells were differentiated under hindbrain conditions until D4 and subject to either FGF/WNT treatment (Figure 5E, H^+^ condition) or FGF/TAM treatment to induce CDX2 (Figure 5E, Hrep condition) between D4 and D5. Strikingly, in contrast to FGF/WNT treatment, which failed to promote posterior identity, the induction of CDX2 led to the repression of PHOX2B cranial pMNs and induction of spinal cord *Hox* genes (Figures 5F and 5G). ATAC-seq analysis demonstrated that, upon induction of CDX2 between D4 and D5, hindbrain accessible sites no longer appeared in these cells and, instead, accessibility at spinal cord sites was observed (Figure 5H). We refer to these cells as “hindbrain reprogrammed” (D5Hrep). These data indicate that, unlike FGF/WNT signaling, the induction of CDX2 in hindbrain progenitors is sufficient to caudalize neural cells to a spinal cord fate.

### *In Vivo* Induction of CDX2 Depends on Developmental Timing

The expression of CDX2 in early mouse embryos is restricted to the posterior epiblast and remains restricted to the posterior as the trunk forms and the axis elongates (van den Akker et al., 2002, Mallo et al., 2010). The *in vitro* results predicted that CDX2 induction by FGF/WNT signals is dependent on developmental timing. To test this *in vivo*, we performed whole mouse embryo culture from epiblast stages (E7.0) and early head-fold stages (E7.5) in the presence of FGF or FGF/WNT signals. Mouse embryos cultured in media with or without added FGF from E7.0 displayed a restricted pattern of WNT signaling in the posterior epiblast (Figure 5I; n = 11/11), similar to WT embryos (Ferrer-Vaquer et al., 2010), and CDX2 expression was detected only in the posterior epiblast (Figure 5I; n = 10/10), as previously shown (Deschamps and van Nes, 2005, Mallo et al., 2010). By contrast, embryos cultured for 12 hr in the presence of FGF/WNT or WNT signals showed ectopic WNT signaling (n = 10/13; Figure 5I) and a marked expansion of CDX2 expression into the anterior epiblast (Figure 5I; n = 10/13). Thus, similar to D2 cells *in vitro*, mouse epiblast tissue is competent to induce CDX2 expression in response to WNT signaling. By contrast, embryos cultured from E7.5, where SOX1 expression demarcates the developing neural plate (Figure 5J), failed to ectopically expand CDX2 expression in the anterior neural plate (n = 31/31 with no expansion), despite ectopic WNT activity in these embryos (n = 13/16; Table S3; Figure 5J). We found a similar stage-specific requirement for FGF/WNT signaling in chick (Figure 5K). Together, these data demonstrate that, similar to *in vitro*, posteriorization *in vivo* is dependent on exposure to FGF/WNT signals in the epiblast, as the competency to upregulate CDX ceases following the establishment of neural identity.

## Discussion

**Figure 6:**
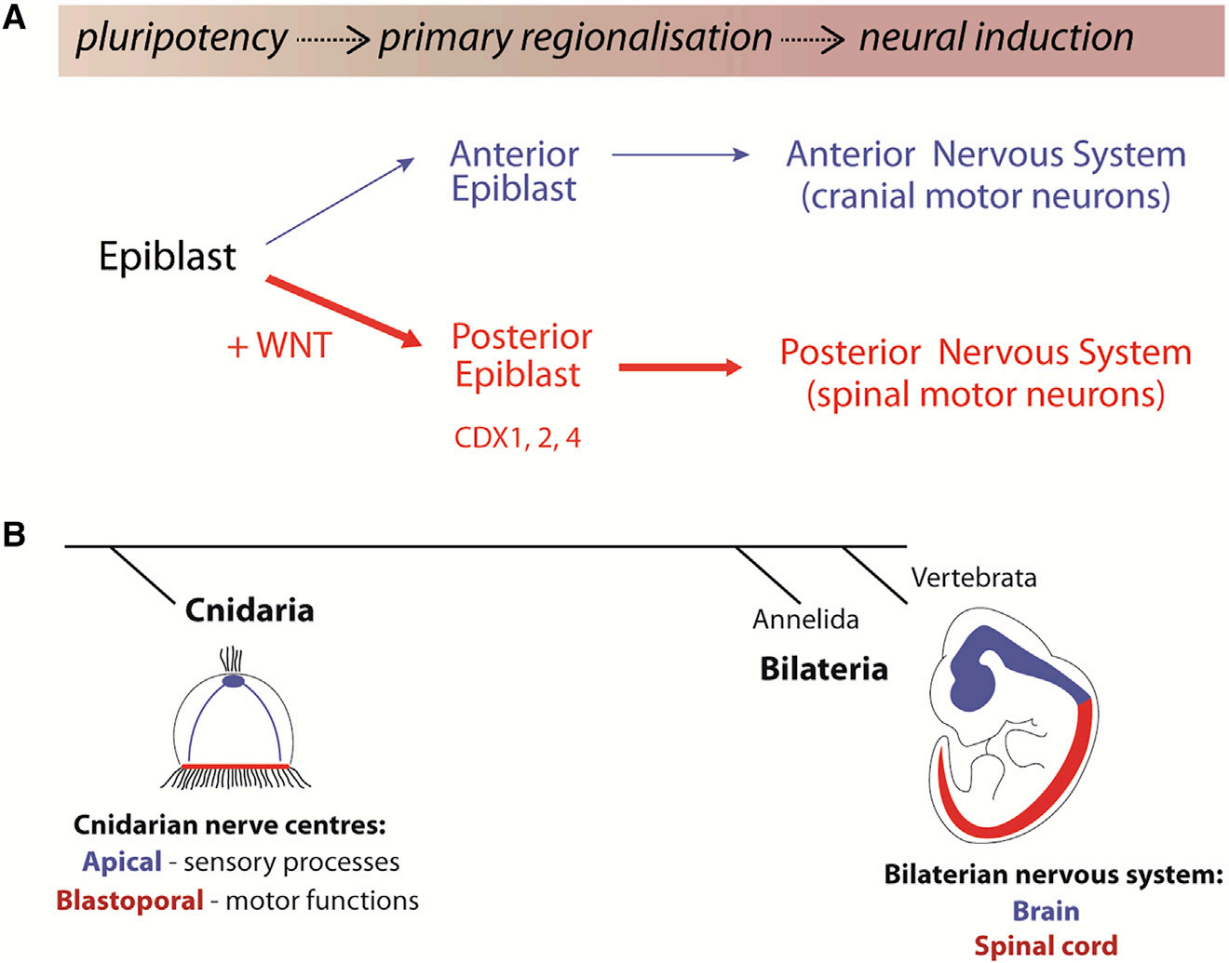
Proposed Model of Nervous System Development (A) Pluripotent epiblast cells in the early embryo are first allocated into anterior (blue) or posterior (red) populations before acquiring neural identity. Posteriorized cells form spinal cord; anterior epiblast cells generate the anterior nervous system. (B) Comparisons between cnidarian and bilaterian animals provide support for the dual evolutionary origin of the vertebrate CNS (Arendt et al., 2016). Cnidarians display two distinct nerve centers: apical (blue) and blastoporal (red). Blastoporal centers show expression of putative CDX orthologs (Arendt et al., 2016, Ryan et al., 2007). In bilaterians, these separate nerve centers are proposed to have expanded and merged.

We provide evidence that the acquisition of spinal cord fate involves cells committing to an axial identity prior to neural induction, reversing the sequence of events implied by the activation-transformation hypothesis (Nieuwkoop, 1952, Stern, 2001) and prompting a revision in our understanding of nervous system regionalization (Figure 6). Support for the activation-transformation hypothesis originated from experiments in chick and frog embryos. For example, explants of posterior axial tissue promoted midbrain and hindbrain fates from prospective forebrain tissue (Cox and Hemmati-Brivanlou, 1995), and manipulating WNT, FGF, and/or RA signaling in neural plate explants altered rostral-caudal identity of neural cells in ways consistent with a graded caudalizing activity (Kolm et al., 1997, Lamb and Harland, 1995, Muhr et al., 1999, Nordström et al., 2006, Wilson et al., 2001). In many of these studies, the most caudal markers assayed were representative of the hindbrain or anterior spinal cord and the results were extrapolated to apply to the entire spinal cord. Although RA exposure to NPs is sufficient to posteriorize anterior neural cells to form hindbrain, the most caudal identity generated in these assays corresponds to cervical (anterior) spinal cord (Gouti et al., 2014, Liu et al., 2001, Mahony et al., 2011, Mazzoni et al., 2013, Niederreither et al., 2000). Furthermore, treatment of anterior NPs with increasing concentrations of WNT fails to caudalize these cells to a spinal cord fate; instead, their identity corresponds to the posterior hindbrain. Thus, the activation-transformation hypothesis seems compatible with regionalization of the forebrain, midbrain, and hindbrain, but not the spinal cord.

The chromatin signatures that define the hindbrain and spinal cord appear simultaneously during neural progenitor differentiation in cells exposed to the same amounts of RA/SHH (Figure 3). Thus, the distinction between regional identities must be established during the preceding period when spinal cord, but not the hindbrain, fated cells receive FGF/WNT signaling. Delaying addition of FGF/WNT signals until after neural identity is established is not sufficient to convert hindbrain cells to a spinal cord identity. Thus, FGF/WNT signaling establishes a posterior program in cells—a “primary regionalization”—before neural induction (Figure 6).

**Figure S5:**
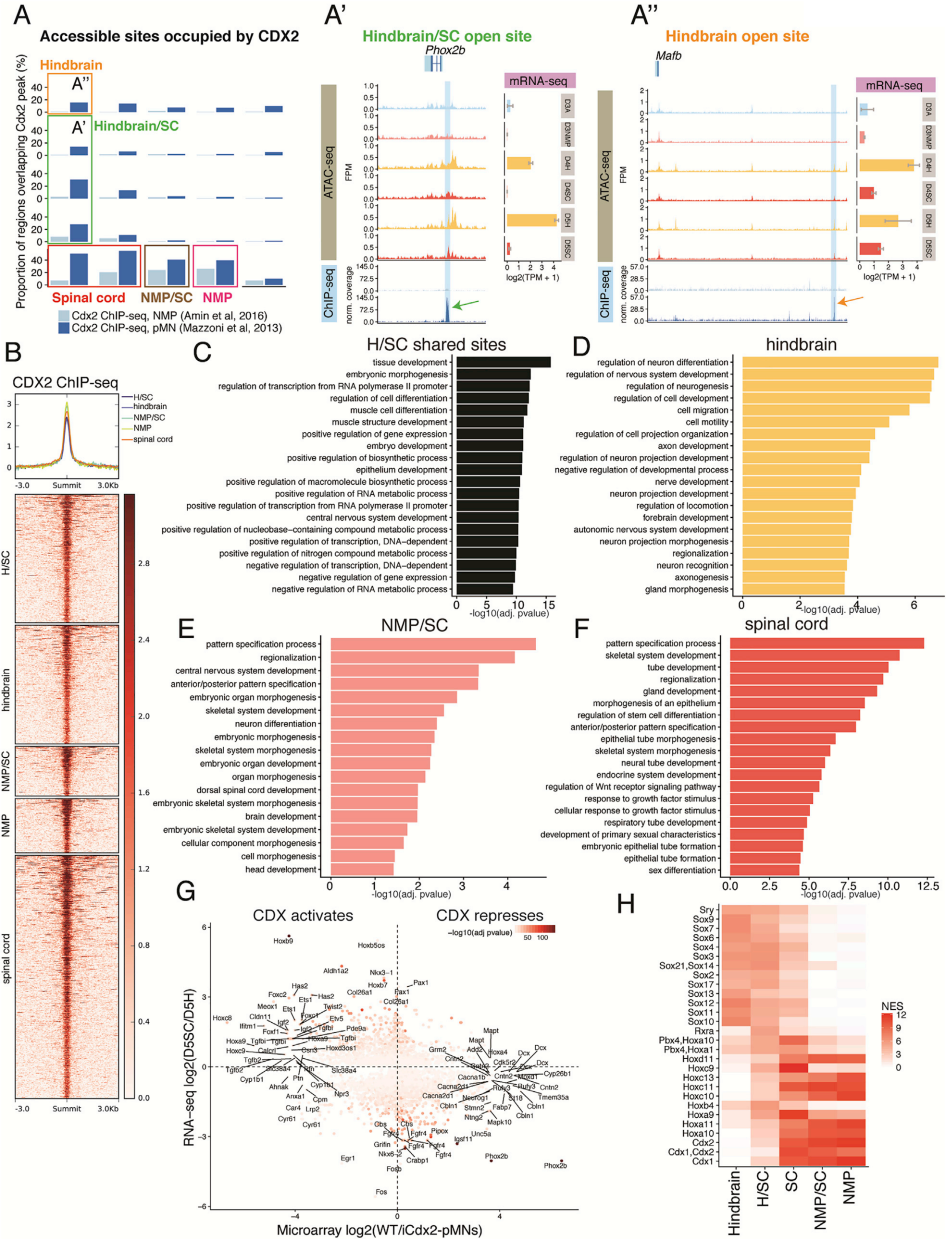
CDX2 Occupancy in Open Chromatin Sites and Associated Gene Ontology Enrichment, Related to Figure 4 (A) The proportion of accessible regions bound by CDX2, as indicated by CDX2 ChIP-seq analysis from neuromesodermal progenitors (NMP, light blue bars, Amin et al., 2016) and motor neuron progenitors (pMNs, dark blue bars, Mazzoni et al., 2013) derived *in vitro*, compared with the accessible regions recovered from the self-organizing map (SOM) in this study (refer to Figure 2A). The overlap demonstrates that CDX2 binds to NMP, NMP/SC (NMP and spinal cord shared) and spinal cord (SC) sites identified by ATAC-seq. Furthermore, in pMN conditions, CDX2 binds accessible regions that are shared between the hindbrain and spinal cord (A’, boxed region outlined in green). CDX2 also targets hindbrain accessible sites (A’’). (A’) The *Phox2b* genomic region represents a shared hindbrain/spinal cord accessible site that is bound by CDX2 in pMN conditions. (A’’) A hindbrain-accessible site is bound by CDX2 at the *Mafb* locus in pMN conditions. (B) Region heatmap showing CDX2 binding at open chromatin sites recovered from the SOM (pMN; Mazzoni et al., 2013). (C–F) Gene ontology enrichment analysis for CDX2-bound regions shown in (B). In hindbrain accessible regions (D), CDX2 binding is associated with neural genes in contrast to either the NMP and spinal cord (NMP/SC) shared (E) or SC-specific sites (F), which target genes involved in anterior-posterior patterning. (G) Comparison of log2 fold gene expression changes in D5 spinal cord (D5SC) versus D5 hindbrain (D5H), determined by mRNA-seq (Gouti et al., 2014), and wild-type (WT) versus *Cdx*2-induced motor neuron progenitors (iCdx2-pMNs) determined by microarray (Mazzoni et al., 2013). CDX2 induction positively correlates with *Hoxb9* and other 5′ *Hox* genes while it negatively correlates with *Aldh1a2* in the spinal cord, in agreement with previous studies (Gouti et al., 2017). CDX negatively correlates with hindbrain genes including *Phox2b*. Color filling indicates –log10(adj. pvalue) from the D5SC versus D5H comparison using DESeq2 (Love et al., 2014). (H) Motif enrichment analysis of CDX2-bound regions shown in (B). Heatmap colors represent the normalized enrichment score computed using iCis Target (Imrichová et al., 2015). CDX2 binds to hindbrain accessible regions that are enriched with SOX factor motifs, in contrast to HOX motifs found in spinal cord sites.

The chromatin regions that responded to FGF/WNT at D3 (Figure 2) were enriched in CDX TF binding sites (Figure 4), consistent with the established role of CDX in promoting posterior embryonic development (Amin et al., 2016, Neijts et al., 2016, Skromne et al., 2007, Young et al., 2009). In addition, CDX2 appears to directly repress genes involved in hindbrain neural identity (Figure S5), indicating a pivotal function for CDX in securing spinal cord identity. The analysis suggests that CDX operates within distinct TF complexes at different target genes (Figure S5H). CDX activity substitutes for WNT signaling to specify spinal cord fate (Figure 5). Moreover, CDX, unlike WNT signaling, reprograms hindbrain cells to spinal cord (Figure 5). This demonstrates the limited developmental competence window, between pluripotent epiblast cells and NPs, in which FGF/WNT signals posteriorize cells. Molecularly, this corresponds to the ability of WNT signaling to induce CDX factors that remodel the chromatin landscape. Hence, a major anterior-posterior division of the nervous system, separating the spinal cord from more rostral territories, is established prior to the acquisition of neural identity by the chromatin remodeling activities of CDX TFs.

The divergence in the mechanisms for formation of the anterior and posterior nervous system is reminiscent of older ideas, in which separate organizers were proposed to induce different parts of the CNS (Mangold, 1933). This is consistent with experiments in chick, in which the regional identity of neural tissue induced by organizer grafts depended on its embryonic age (Storey et al., 1992), and the observation that hindbrain tissue transplanted into the spinal cord does not readily adopt a spinal cord identity (Grapin-Botton et al., 1997). Moreover, AP patterning events in the epiblast are thought to be distinct from the neural inducing activities of the organizer in fish (Koshida et al., 1998).

The separate lineages generating hindbrain and spinal cord prefigure differences in the complement of neurons generated in these regions (Carcagno et al., 2014, Cordes, 2001, Shirasaki and Pfaff, 2002). Primary regionalization in the precursors that generate trunk and cranial cell types may contribute to these differences (Frith et al., 2018). The finding that regionalization is initiated and differences established prior to neural induction highlight the importance of determining the appropriate conditions for the directed differentiation of ESCs into defined neural fates.

The role of the WNT-CDX genetic network in the specification of caudal tissue has been documented across the bilaterian clade (Faas and Isaacs, 2009, Morales et al., 1996). This broad evolutionary conservation implicates a functional role for this network in the last common bilaterian ancestor (Ryan et al., 2007). The divergent lineage and distinct molecular events of the anterior and posterior nervous system are consistent with the proposed dual evolutionary origins of the CNS (Arendt et al., 2016). This hypothesis postulates that the bilaterian nervous system arose from the merger of nerve centers residing at opposite poles of the ancestral pre-bilaterian animal (Arendt et al., 2016). The expansion and fusion of these then led to the bilaterian nerve cord and brain (Arendt et al., 2016, Tosches and Arendt, 2013). Hence, the distinct molecular mechanisms that specify anterior versus posterior vertebrate nervous systems may represent an evolutionary vestige of the processes that once generated neural tissue in pre-bilaterian animals.

## STAR★Methods

### Key Resources Table

**Table:** 

REAGENT or RESOURCE	SOURCE	IDENTIFIER
**Antibodies**		

Mouse monoclonal anti-CDX2	Abcam	157524; RRID:AB_2721036
Goat polyclonal anti-SOX2	R&D	AF2018; RRID:AB_355110
Goat polyclonal anti-SOX1	R&D	AF3369; RRID:AB_2239879
Rabbit polyclonal anti-SOX2	Cell Signaling	2748S; RRID:AB_823640
Rabbit polyclonal anti-PHOX2B	Jean-Francois Brunet Lab	RRID:AB_2313690
Rabbit polyclonal anti-OLIG2	Millipore	AB9610; RRID:AB_570666
Guinea pig polyclonal anti-OLIG2	Ben Novitch Lab	RRID:AB_2715520
Anti-DIG-AP antibody	Roche	11093274910; RRID:AB_514497
Mouse monoclonal anti-CDX2-PE	BD	563428; RRID: AB_2738198
Mouse monoclonal anti-SOX2-v450	BD	561610; RRID: AB_10712763
Goat polyclonal anti-SOX2	Santa Cruz	SC-17320X; RRID:AB_2286684

**Chemicals, Peptides, and Recombinant Proteins**		

bFGF	R&D	3139-FB
CHIR99021	Axon	1386

**Critical Commercial Assays**		

Nextera DNA library prep kit	Illumina	FC-121-1030
Kapa Hyper Prep Kit	Illumina	KK8502
In-Fusion Cloning Kit	Takara	639650
Phusion High-Fidelity PCR Master Mix	ThermoFisher Scientific	F532L

**Deposited Data**		

ATAC-seq on ESCs undergoing directed differentiation toward defined neural fates	This paper	ArrayExpress accession E-MTAB-6337
Sox2 ChIP-seq in hindbrain and spinal cord neural progenitors differentiated from mouse embryonic stem cells	This paper	ArrayExpress accession E-MTAB-6348

**Experimental Models: Cell Lines**		

HM1 mESCs	Thermo Scientific, MES4303	Doetschman et al., 1987
*T/Bra*^*−/−*^ mESCs	James Briscoe Lab	Gouti et al., 2017
*Cdx*^*1,2,4−/−*^ mESCs	James Briscoe Lab	Gouti et al., 2017
iCDX2 ESCs	Hitoshi Niwa (provided by Myriam Hemberger, Babraham Institute)	5ECER4G20; Niwa et al., 2005

**Experimental Models: Organisms/Strains**		

Mouse: *Sox2eGFP*	Robin Lovell-Badge Lab	MGI:3589809, Ellis et al., 2004
Mouse: *TCF/Lef:H2B-mCherry*	This paper	N/A
Chicken: Dekalb strain	Henry Stewart & Co.	N/A

**Oligonucleotides**		

Primers for RT-qPCR	This paper	Table S5
Primer: mCherry forward: ATGGTGAGCA AGGGCGAG	This paper	N/A
Primer: mCherry reverse: CTTGTACAGC TCGTCCATGC	This paper	N/A
Primers for indexing ATAC-seq libraries	Buenrostro et al., 2013	Table S6
Primer: TCF/Lef:H2B:mCherry cloning forward: GGGCTCTAGGGATCCATCGATGGGAACA AAAGCTGGTACCGG	This paper	N/A
Primer: TCF/Lef:H2B:mCherry cloning reverse: GGTACCCGGGGATCCATCGATCGCTTACA ATTTACGCCTTAAGATACATTGATGAG	This paper	N/A

**Recombinant DNA**		

Chicken *Cdx2* RNA *in situ* hybridization probe	Source Bioscience	EST Clone ChEST626o23
pJC5-4	Chung et al., 1993	N/A
T*cf.*/Lef:H2B-GFP	Addgene	32610

**Software and Algorithms**		

All data analysis scripts used in this paper	This paper	https://github.com/luslab/NeuralATACseq
Fiji	Schindelin et al., 2012	http://fiji.sc/
GraphPad Prism 7	GraphPad	https://www.graphpad.com/
Trim Galore! v0.4.1	N/A	https://www.bioinformatics.babraham.ac.uk/projects/trim_galore/
Bowtie2 v2.3.2	Langmead and Salzberg, 2012	https://sourceforge.net/projects/bowtie-bio/files/bowtie2/
Piccard v2.9.2	N/A	https://broadinstitute.github.io/picard/
DeepTools v3.0.0	Ramírez et al., 2016	https://deeptools.readthedocs.io/en/develop/
MACS2 v2.1.0	Zhang et al., 2008	https://github.com/taoliu/MACS/
IDR framework	Li et al., 2011	https://sites.google.com/site/anshulkundaje/projects/idr/deprecated
BEDTools v2.26.0	Quinlan and Hall, 2010	https://bedtools.readthedocs.io/en/latest/
Subread v1.5.0	Liao et al., 2013	http://subread.sourceforge.net/
GREAT v3.0.0	McLean et al., 2010	http://great.stanford.edu/public/html/index.php
HOMER v4.9.1	Heinz et al., 2010	http://homer.ucsd.edu/homer/motif/
i-cisTarget 2015	Imrichová et al., 2015	https://gbiomed.kuleuven.be/apps/lcb/i-cisTarget/
R v3.4.0	R Foundation	https://www.r-project.org/
DESeq2 v1.18.1	Love et al., 2014	https://bioconductor.org/packages/release/bioc/html/DESeq2.html
SOM v0.3-5.1	N/A	https://cran.r-project.org/web/packages/som/som.pdf
CENTIPEDE v1.2	Pique-Regi et al., 2011	http://centipede.uchicago.edu/
GenomicRanges v1.30.3	Lawrence et al., 2013	https://bioconductor.org/packages/release/bioc/html/GenomicRanges.html
LOLA v1.8.0	Sheffield and Bock, 2016	http://bioconductor.org/packages/release/bioc/html/LOLA.html
Motifmatchr v1.0.1	N/A	https://github.com/GreenleafLab/motifmatchr
ChIPseeker v1.14.2	Yu et al., 2015	https://bioconductor.org/packages/release/bioc/html/ChIPseeker.html
Limma v3.34.3	Ritchie et al., 2015	https://bioconductor.org/packages/release/bioc/html/limma.html
Oligo v1.42.0	Carvalho and Irizarry, 2010	https://bioconductor.org/packages/release/bioc/html/oligo.html

**Other**		

ENCODE data portal	ENCODE Project Consortium, 2012, Sloan et al., 2016	https://www.encodeproject.org/
JASPAR database	Khan et al., 2018	http://jaspar.genereg.net/
4DGenome database	Teng et al., 2015	https://4dgenome.research.chop.edu/
GENCODE release M14	Mudge and Harrow, 2015	https://www.gencodegenes.org/mouse_releases/14.html

### Contact for Reagent and Resource Sharing

Further information and requests for resources and reagents should be directed to and will be fulfilled by the Lead Contact, James Briscoe (james.briscoe@crick.ac.uk)

### Experimental Model and Subject Details

All animal procedures were performed in accordance with the Animal (Scientific Procedures) Act 1986 under the UK Home Office project licenses PPL80/2528 and PD415DD17.

#### Sox2eGFP mouse line

*Sox2eGFP* heterozygous mice (Ellis et al., 2004) were maintained on a C57BL6 background. To harvest embryos at E9.5, *Sox2eGFP* heterozygous mice were time-mated to wild-type C57BL6 mice.

#### TCF/Lef:H2B:mCherry WNT reporter line

The 6xTCF/Lef:H2B:mCherry sequence (originally from Addgene, #32610, GFP replaced by mCherry) was cloned using In-Fusion Cloning (Takara, 639650) between two chicken insulators at the BamHI site of plasmid pJC5-4 (Chung et al., 1993) without the LCR fragment. The 2.6Kb 6x TCF/Lef:H2B:mCherry fragment was amplified using Phusion High-Fidelity PCR Master Mix (ThermoFisher Scientific, F532L) according to the manufacturer’s instructions. The final plasmid was linearized with NdeI/ScaI and used for pronuclear injection using fertilized embryos from the F1 hybrid strain (C57BL6/CBA). Mice with WNT reporter activity as previously described (Ferrer-Vaquer et al., 2010) were verified by genotyping using primers that detect the mCherry fragment. The WNT reporter line was maintained on the F1 background by crossing heterozygous *TCF/Lef:H2B:mCherry* mice to F1 wild-type mice.

#### Cell lines

All cell lines (XY male) were maintained and experiments performed at 37°C with 5% carbon dioxide (CO_2_). All WT ESC culture was performed using the HM1 line (Doetschman et al., 1987). *Bra*^*−/−*^ and *Cdx*^*1,2,4−/−*^ knockout ESC lines were generated in the HM1 line using CRISPR as previously described (Gouti et al., 2017). Single guide RNAs were used to target the T-box domain (*T/Bra* mutant), and the caudal-like activation domain of *Cdx1*, *Cdx2* and *Cdx4* (*Cdx*^*1,2,4−/−*^ triple mutant). All iCDX2 ESC experiments were performed using the 5ECER4G20 ESCs (Niwa et al., 2005). Cell lines were validated by DNA sequencing and western blotting and routinely tested for mycoplasma.

### Method Details

#### Mouse whole embryo culture

Embryos were dissected at E7.0 (early to mid-streak stage) and E7.5 (neural plate stage-early head fold stage) in pre-equilibrated DMEM supplemented with 10% fetal bovine serum, 25 mM HEPES-NaOH (pH 7.2), containing penicillin/streptomycin (GIBCO). Extraembryonic and visceral endoderm tissues (for E6.5 embryos) were removed. Embryos were then transferred into 75% fresh rat serum, 25% DMEM without phenol red, 2nM L-Glutamine. Control embryos were cultured in medium with or without 10ng/ml bFGF and displayed no differences in CDX2 expansion (see Table S3). Treated embryos were cultured with the same media with additional 20 μM CHIR99021 (Axon), similar to previous studies (Neijts et al., 2016), with or without 10ng/ml bFGF (Peprotech; see Table S3). Embryos were cultured in Mattek dishes (static culture) for 12-14 hours in 5%O_2_, 5%CO_2_, 90%N_2_ (for E7.0 embryos) or 5%CO_2_, 95%0_2_ (for E7.5 embryos) at 37°C.

#### Wholemount immunofluorescence and image acquisition

Cultured embryos were fixed for 20min (for early E7.0 cultured embryos) or overnight (for late E7.5 cultured embryos) in 2% PFA at 4°C, then permeabilized in PBST (PBS containing 0.5% Triton X-100) for 15min and blocked (5% donkey serum/5% BSA). Embryos were incubated overnight at 4°C with antibodies diluted in PBST (PBS 0.1% Triton X-100): mouse anti-CDX2 (1:250, abcam 157524; RRID:AB_2721036), goat anti-SOX2 (1:250, R&D Systems, AF2018; RRID:AB_355110) or goat anti-SOX1 (1:250, R&D Systems, AF3369; RRID:AB_2239879). After washing in freshly prepared PBST at 4°C, embryos were incubated with secondary antibodies (Molecular Probes) coupled to AlexaFluor 488 or 647 fluorophores as required at 1:250 overnight at 4°C. Before imaging, embryos were washed in PBST at room temperature. Confocal images were obtained on an inverted Zeiss 710 confocal microscope with a 10X air objective (0.4 NA) at a 2048 × 2048 pixels dimension with a z-step of 6-7 μm (2 × 1 tile scale, for the late E7.5 cultured embryos). Embryos were systematically imaged throughout from top to bottom. Images were processed using Fiji software (Schindelin et al., 2012).

#### Chick whole embryo culture

Chick embryos at Hamburger Hamilton stage (HH st) 3 - 3+ or older were isolated from the yolks using forceps and scissors and transferred in PBS using a perforated spoon. Embryos were subsequently transferred to a Petri dish containing Leibovitz’s (L15) tissue culture medium and blastoderms were freed gently from the membranes using fine forceps. The isolated blastoderms were transferred to a fresh Petri dish containing L15 and kept on ice until a sufficient number of blastomeres had been collected for each experiment. To prepare embryos for culture (Connolly et al., 1995), each blastoderm was turned hypoblast side up and any large lumps of yolk removed with fine forceps. Disk-shaped blastoderms were held with forceps on one side and folded over along the longitudinal axis of the embryo to form a “Cornish pasty” shape, where the hypoblast/endoderm lay inside and the ectoderm outside. The free edges were sealed by pinching with fine forceps followed by cutting with iridectomy scissors along a line just within the area opaca. Folded and sealed embryos were transferred to 5ml bijou bottle containing 1ml of 10% FBS, 100U/ml Penicillin-Streptomycin in L15. A maximum of 5 embryos were cultured per bottle. The bottles were placed in a roller incubator (BTC Engineering) at 38°C for 10 hours. Control embryos were cultured in media alone or in the presence of 10ng/ml bFGF and displayed no differences in *Cdx2* expansion. Treated embryos were cultured with 10ng/ml bFGF (Peprotech) and 10 μM CHIR99021 (Axon). All chicken embryos were supplied by Henry Stewart & Co.

#### Chick wholemount *in* *situ* hybridization and image acquisition

The chick *Cdx2* DIG labeled RNA riboprobe was synthesized using the EST Clone ChEST626o23 as a template (Source Bioscience). Antisense riboprobe was linearized using NotI and transcribed with T3 RNA polymerase. For *in situ* hybridization (ISH), embryos were fixed in 4%PFA/PBS overnight at 4°C, then dehydrated in a PBT/methanol series and stored in 100% methanol (−20°C). Embryos were rehydrated in a PBT/methanol series followed by 1h treatment with 6% hydrogen peroxide, 5-10min proteinase K treatment (10μg/ml), glycine washes and fixation in 4%PFA/PBS, 0.2% gluteraldehyde. Prehybridization was performed in 50% formamide, 5xSSC, 0.1% Tween20, 100μg/ml Heparin Sodium Salt at 65°C for 1-2 hours. Hybridization was performed in hybridization buffer (50% formamide, 5xSSC, 0.1%Tween20, 100ug/ml Heparin Sodium Salt, 0.1mg/ml Torula yeast RNA, 0.1mg/ml herring sperm DNA) containing 1μg/ml DIG labeled RNA probe at 68°C, O/N. Post hybridization, the embryos were washed in 50% formamide, 4x SSC, 1%SDS (69°C, 1h), treated with RNase A (37°C, 1h), washed in 50% formamide, 2x SSC (65°C, 30min), washed in TBST and blocked with 10% sheep serum for 1-2h. Anti-DIG-AP antibody (Roche, 11093274910) was then used at 1/2000 dilution in 1% sheep serum TBST (4°C, O/N). The following day the embryos were washed thoroughly in TBST, pH was increased to pH9 in NTMT buffer and the staining developed in the presence of NBT-BCIP for 1-2h at room temperature.

#### Cell culture and neural progenitor differentiation

All mouse ESCs were propagated on mitotically inactivated mouse embryonic fibroblasts (feeders) in DMEM knockout medium supplemented with 1000U/ml LIF (Chemicon), 10% cell-culture validated fetal bovine serum, penicillin/streptomycin, 2mM L-glutamine (GIBCO). To obtain neural progenitors with anterior, hindbrain or posterior neural identity, ESCs were differentiated as previously described (Gouti et al., 2014). Briefly, ESCs were dissociated with 0.05% trypsin, and plated on gelatin-coated plates for two sequential 20-minute periods in ESC medium to separate them from their feeder layer cells which adhere to the plastic. To start the differentiation, cells remaining in the supernatant were pelleted by centrifugation, washed in PBS, and pelleted again. Cells were counted and resuspended in N2B27 medium containing 10ng/ml bFGF to a concentration of 10^6^ cells per ml, and 50,000 cells per 35mm CELLBIND dish (Corning) were plated. N2B27 medium contained a 1:1 ratio of DMEM/F12:Neurobasal medium (GIBCO) supplemented with 1xN2 (GIBCO), 1xB27 (GIBCO), 2mM L-glutamine (GIBCO), 40μg/ml BSA (Sigma), penicillin/streptomycin and 0.1mM 2-mercaptoethanol. To generate anterior neural progenitors, the cells were grown up to day (D) 3 in N2B27 + 10ng/ml bFGF, followed by N2B27 + 500nM smoothened agonist (SAG; Calbiochem) from D3-5. To generate hindbrain neural progenitors, cells were cultured under the same conditions as the anterior, but were additionally exposed to 100nM retinoic acid (RA; Sigma) from D3-5. To generate spinal cord neural progenitors, cells were cultured with N2B27 + 10ng/ml bFGF until D2, N2B27 + 10ng/ml bFGF + 5μM CHIR99021 (Axon) until D3, and N2B27 + 100nM RA + 500nM SAG until D5. For Hindbrain+ treated cells (Figure 3), cells were differentiated under hindbrain conditions with one modification between D4-5, where they were additionally exposed to 10ng/ml bFGF and 5μM CHIR99021 in addition to continued treatment with RA and SAG as above. For the inducible CDX2 experiments (Figure 5), induction of CDX2 was performed by adding 1μg/ml of 4-hydroxy tamoxifen (Sigma) (Niwa et al., 2005) between D2-3 (Figure 5A; in the presence of bFGF in N2B27) or D4-5 (Figure 5E; in the presence of 10ng/ml bFGF/100nM RA/500nM SHH). For all differentiations, media changes were made every 24 hours from D2. All experiments were performed using biological triplicates.

#### Immunofluorescence and microscopy on cells

Cells were washed in PBS and fixed in 4% paraformaldehyde in PBS for 15min at 4°C, followed by two washes in PBS and one wash in PBST (0.1% Triton X-100 diluted in PBS). Primary antibodies were applied overnight at 4°C diluted in filter-sterilized blocking solution (1% BSA diluted in PBST). Cells were washed 3x in PBST and secondary antibodies (AlexaFluor conjugated; Invitrogen) were applied at room temperature, diluted 1:1000 in PBS for 1hr. Cells were washed 3x in PBS, incubated with DAPI for 5 min in PBS and washed twice before mounting with Prolong Gold (Invitrogen). Primary antibodies were diluted as follows: mouse anti-CDX2 (1:250, Abcam 157524, RRID:AB_2721036), rabbit anti-SOX2 (1:500, Cell Signaling 2748S, RRID:AB_823640) and goat anti-SOX1 (1:250, R&D AF3369, RRID:AB_2239879); PHOX2B rabbit, kindly provided by Jean-Francois Brunet (Pattyn et al., 1997), RRID:AB_2313690, 1/1000; OLIG2 rabbit (Millipore, AB9610; RRID:AB_570666; 1/1000); Olig2 guinea-pig, kindly provided by Ben Novitch (Novitch et al., 2001), RRID:AB_2715520, 1/10000, and SOX2 goat (R&D Systems, AF2018; RRID:AB_355110; 1/500). Cells were imaged on a Zeiss Imager.Z2 microscope using the ApoTome.2 structured illumination platform. Z stacks were acquired and represented as maximum intensity projections using ImageJ software. To perform confocal imaging (Figure 4K and S4D), cells were differentiated on glass-like chamber slides (LabTek, 177437) that were pre-coated for 1hr at room temperature with 1:25 dilution of Matrigel (Corning, 356231), diluted in DMEM/F12 media (GIBCO) to facilitate adherence. Cells were imaged using a Leica SP5 confocal microscope with a 40x oil immersion objective at a at a 1024 × 1024 pixel dimension. Single plane confocal images were acquired under identical conditions. Pixel intensities were adjusted across the entire image in Fiji. The same settings were applied to all images. Immunofluorescence was performed on a minimum of 3 biological replicates, from independent experiments.

#### Intracellular Flow Cytometry

Cells were washed in PBS and dissociated with 0.5ml accutase (GIBCO). Once detached cells were collected into 1.5mL Eppendorf tubes, plates washed once with N2B27 and pelleted. Cells were resuspended in PBS, pelleted and resuspended in 4% paraformaldehyde in PBS. Following a 20min incubation step at 4°C, cells were centrifuged, resuspended in 1mL PBS, and stored at 4°C for future analysis. On the day of flow cytometry, cells were counted and 2x10^6^ aliquoted per sample into 1.5mL Eppendorf tubes for staining. Samples were pelleted and resuspended in 100uL PBST + 0.1% BSA. After a 45min incubation at room temperature an antibody mix of CDX2-PE (BD, 563428,1:50), SOX2-v450 (BD, 561610, 1:50) was added to the sample and incubated at room temperature for a further 45min. Cells were pelleted at 2,000rpm for 5min and resuspended in 0.5mL PBS. One additional wash was performed before acquisition on an LSR Fortessa (BD). Analysis was performed using FlowJo software. Cells were first gated on SOX2 before plotting CDX2 intensities. CDX2 positive cells were defined using the *Cdx* triple knockout cell line as a negative control for each time point and condition.

#### RNA extraction, cDNA synthesis and qPCR analysis

RNA was extracted from cells using a QIAGEN RNeasy kit, following the manufacturer’s instructions. Extracts were digested with DNase I to eliminate genomic DNA. First strand cDNA synthesis was performed using Superscript III (Invitrogen) using random hexamers and was amplified using Platinum SYBR-Green (Invitrogen). qPCR was performed using the Applied Biosystems 7900HT Fast Real Time PCR. PCR primers were designed using NCBI primer blast or primer3 software, using exon-spanning junctions where possible. Expression values for each gene were normalized against β-actin, using the delta-delta CT method. Error bars represent standard deviation across three biological replicate samples. qPCR was performed on 3 biological replicates for every primer pair analyzed. Primer sequences are available in Table S5.

#### ATAC-seq

ATAC-seq was performed following methods previously described (Buenrostro et al., 2013). Adherent cells were treated with StemPro Accutase (A1110501) to obtain a single cell suspension. Cells were counted and resuspended to obtain 50,000 cells per sample in ice-cold PBS. Cells were pelleted and resuspended in lysis buffer (10mM Tris-HCl pH 7.4, 10mM NaCl, 3mM MgCl_2_, 0.1% IGEPAL). Following a 10min centrifugation at 4°C, nucleic extracts were resuspended in transposition buffer for 30min at 37°C and purified using a QIAGEN MinElute PCR Purification kit following manufacturer’s instructions. Transposed DNA was eluted in a 10μL volume and amplified by PCR with Nextera primers (Buenrostro et al., 2013) to generate single-indexed libraries. A maximum of 12 cycles of PCR was used to prevent saturation biases based on optimization experiments performed using qPCR. Library quality control was carried out using the Bioanalyzer High-Sensitivity DNA analysis kit. Libraries were sequenced as paired-end 50 or 100 bp reads, multiplexing 4 samples per lane on the Illumina High-Seq 2500 platform at the Francis Crick Institute Advanced Sequencing Facility. For all conditions, two biological replicate samples were collected from independent experiments.

#### In vivo ATAC-seq

Embryos for ATAC-seq were harvested at E9.5 in HBSS buffer (GIBCO) containing 5% FBS. As the ratio of cells to transposase is a critical parameter in generating ATAC-seq results (Buenrostro et al., 2015), we aimed to use the same ratio of cells *in vivo* as *in vitro* for maximum comparability. To obtain sufficient quantities of cells from *in vivo*, *Sox2eGFP* positive embryos (described above) from several litters were pooled together and screened for GFP using a Leica MZFL widefield microscope with a GFP filter set. Embryos were separated into GFP positive and negative pools. To enrich for anterior (forebrain, midbrain, and/or anterior hindbrain) and spinal cord neural progenitors, GFP positive embryos were dissected as follows: heads were decapitated at the second pharyngeal arch and otocysts removed to avoid contamination with other *GFP*-expressing cells. To obtain spinal cord NPs, the neural tube and surrounding somitic tissue was dissected, from the level of caudal hindbrain to the tailbud posterior neuropore. Both cranial and trunk regions were minced with forceps, incubated for 5min on ice in enzyme-free dissociation buffer (GIBCO) and then gently passed through a 40μm filter using the plunger from a sterile syringe. Dissociated cells were collected, centrifuged at 4°C for 5min at 1500 rpm and resuspended in 500ul HBSS buffer containing 5% FBS. Cells were passed through a 40μm filter and sorted using flow cytometry. Flow analysis and sorting was performed by the Francis Crick Flow Cytometry facility, using an Aria Fusion cell sorter with a 488nm laser. GFP negative cells (obtained from negative littermates collected in parallel) were used as a negative control to set voltage gating. 50,000 GFP positive cells from anterior and spinal cord levels obtained from FACS were subject to ATAC-seq as described for *in vitro*-derived cells. Duplicate samples were collected on independent days to represent biological repeats.

#### ChIP-seq

Sox2 ChIP-seq was performed using 10-30 million cells from D5 hindbrain and D5 spinal cord neural progenitors. Cells were washed twice in PBS and crosslinked in 1% formaldehyde for 20min at 4°C. 1M glycine was added for 5min at 4°C and cells were washed 2-3 times in PBS at 4°C. Cells were scraped from the culture dish and transferred to a low-binding tube (AM12450) and centrifuged briefly at maximum speed to pellet the cells. Supernatant was removed and cells were snap-frozen in liquid nitrogen. Cells were thawed on ice and resuspended in a maximum volume of 300μL lysis solution containing SDS lysis buffer (Millipore, 20-163), protease inhibitors (Sigma P8340, diluted 1:500) and PMSF (Sigma, 93482, diluted 1:100). Chromatin was sonicated using a Diagenode Bioruptor (using a cycle of 30s on, 30s off) until fragments were between 200-400bp. 3μg SOX2 antibody (SC-17320X) was incubated together with the cell lysate overnight at 4°C on a rotating wheel. Immunoprecipitation of chromatin fragments was captured using Protein G-coupled Dynabeads (Life Technologies). Samples were decrosslinked and purified using the QIAGEN MinElute kit. Approximately 10ng ChIP DNA and 10ng input DNA for each condition was used to prepare ChIP-seq libraries using the KAPA Hyper Prep Kit (Illumina). Biological duplicates were obtained for both conditions from separate experiments. Libraries were sequenced as single-end, 50bp reads on the Illumina High-Seq 2500 platform (Francis Crick Institute).

#### ATAC-seq data pre-processing

For each ATAC-seq sample, sequencing adapters and poor quality bases were trimmed from sequencing reads using trim_galore with default settings (https://www.bioinformatics.babraham.ac.uk/projects/trim_galore/). Reads were mapped to the *mm10* reference genome using bowtie2 (parameters *-X 2000—sensitive-local*) (Langmead and Salzberg, 2012). Unmapped reads, reads mapping at low quality (MAPQ < 30), reads mapping to chrM, and unpaired reads were removed. PCR duplicates were removed using Piccard. Coverage tracks were computed as fragments per million per base pair (FPM) using deepTools bamCoverage (parameters *–scaleFactor 10*^*6*^*/Library size –bs 1 –extendReads –samFlagInclude 66 –ignoreDuplicates*) (Ramírez et al., 2016) and enriched genomic loci were identified using MACS2 (parameters *-g mm –p 0.01 –nomodel –f BAMPE*) (Zhang et al., 2008). For analyses measuring insertion level (e.g., TF footprinting) we shifted plus-strand insertions by +4bp and minus-strand insertions by −5bp (Buenrostro et al., 2013).

For each condition, we combined biological replicates by computing the irreproducible discovery rate (IDR) and thresholding for peaks (IDR ≤ 0.1) (Li et al., 2011). Finally, we defined a consensus peak set for all conditions by merging overlapping peaks across different conditions using BEDTools merge (Quinlan and Hall, 2010). The coverage across the consensus peaks were calculated for each condition using featureCounts (parameters *–F SAF –p –ignoreDup*) (Liao et al., 2013), with higher coverage indicating greater chromatin accessibility.

To identify differentially accessible ATAC-seq peaks between conditions, we used the above count table for the consensus peak set as input data for DESeq2 (default settings); pairwise comparisons were made between D0 and each WT *in vitro* condition (Love et al., 2014). Statistically significant differential peaks were identified as those with log2(FC) > 1 and adjusted p value < 0.01.

#### ATAC-seq – SOM cluster analysis

Differentially accessible ATAC-seq peaks were clustered using a self-organizing map (SOM). For this, the DESeq2 normalized count data for each region was transformed into z-scores (z = (x – mean(x))/sd(x)). The resulting z-score matrix was used as input for the SOM with a 5x5 cell grid, hexagonal topology and Gaussian neighborhood function. We found that 5x5 provides the best separation of differential ATAC-seq peaks, though different grid sizes produced similar results. We classified SOM clusters into the conditions in which the differential peaks were most accessible.

To compare the D5H and D5H+ conditions, we first identified robust peaks in D5H+ and added them to our consensus peak set. Differentially accessible peaks were identified using the same methods as above. Differential peaks were overlapped with the SOM clusters (Figure S4B) and used as input for i-cisTarget motif enrichment (Figure S4C).

#### ATAC-seq - peak annotation

The consensus peaks were assigned to nearby genes using ChIPseeker annotatePeak (Yu et al., 2015) with GENCODE release M14 (Mudge and Harrow, 2015) and GREAT (default settings) (McLean et al., 2010). Each gene was assigned to a regulatory region spanning 5kb upstream and 1kb downstream of the TSS (irrespective of other genes). This regulatory domain was extended in both directions to all nearest genes, up to a maximum of 1000kb (McLean et al., 2010).

#### ATAC-seq – *In vitro* versus *in vivo* comparative data analysis

To compare our D5A and D5SC *in vitro* neural progenitors to *in vivo*-derived progenitors, we mapped the ATAC-seq fragment coverage obtained from the *in vivo* datasets to the consensus peak set using featureCounts with the same parameters as above. We normalized the counts from the two datasets using DESeq2 with default settings. Next, we selected the consensus peaks belonging to the anterior, neural and spinal cord SOM clusters (Figure 2A), and for each condition-pair, we compared the log2 fold-change distribution using a two-sided Wilcoxon rank sum test (Figure 2F). To plot the ATAC-seq meta profiles we included consensus peaks enriched in both *in vitro* and the corresponding *in vivo* sample (abs(log2(FC)) > 0.5).

To compare NMP, NMP/SC and Epi SOM cluster peak sets (Figure 2A) to the *in vivo* datasets (Figure 4A), MACS2 called peaks from E6.0, E7.2, E7.5P (Neijts et al., 2016) and E9.5SC and E9.5A neural progenitors (Figure 2E) were compared to each SOM cluster set by computing the peak overlap using findOverlaps from the GenomicRanges package (Lawrence et al., 2013). The resulting proportion of regions overlapping was calculated relative to the size of the SOM cluster (Figure 4A).

#### ATAC-seq - ENCODE DHS overlap

DNase hypersensitive sites (DHS) in the *mm10* reference genome were obtained from the ENCODE data portal (ENCODE Project Consortium, 2012, Sloan et al., 2016). DHS regions were overlapped with our consensus peak set using GenomicRanges findOverlaps.

#### ATAC-seq - Vista enhancer enrichment

Vista enhancer regions were downloaded from the ENCODE data portal (ENCODE Project Consortium, 2012, Sloan et al., 2016) (https://www.encodeproject.org/). Enhancers were overlapped with our consensus peak set and enhancer enrichments were assessed with a one-sided binomial test (McLean et al., 2010). P values were corrected for multiple testing using the Benjamini-Hochberg procedure.

#### ATAC-seq - motif enrichment

TF binding motif enrichments in the consensus peaks were examined using Homer and i-cisTarget. For Homer, we extracted ± 150bp around the center of each consensus peak using BEDTools *slop* and the resulting FASTA files were used as input for HOMER findMotifs.pl (parameters *–bits –mset vertebrates*) (Heinz et al., 2010).

For i-cisTarget, genomic coordinates for the consensus peaks were mapped from the *mm10* to *mm9* reference genome using UCSC-liftOver. These were then uploaded to the i-cisTarget webserver to perform a *full analysis* with default settings (species = *mm9*) (Imrichová et al., 2015). The resulting motif enrichments were downloaded, filtered for redundant motifs and visualized as heatmaps.

#### ATAC-seq - TF footprinting

TF binding motifs of interest were obtained from the JASPAR database (Khan et al., 2018). We searched for motif matches using motifmatchr (https://github.com/GreenleafLab/motifmatchr). Searches were performed either genome-wide (for CTCF; Figure S1F) or ± 5kbp around consensus peak summits (for CDX; Figure S4D). The resulting matches were extended ± 150bp. An insertion count matrix at base pair resolution, centered on the motifs, was generated by counting adjusted Tn5 insertions. Only fragments smaller than 100bp were considered (nucleosome free fraction).

For the footprinting, we used PWM scores and corresponding insertion count matrix as the input for CENTIPEDE to compute posterior probabilities that a motif is bound (Pique-Regi et al., 2011). A different threshold to classify bound/unbound was used depending on the motif matching strategy (genome-wide: > = 0.99; peak summit: > = 0.9).

#### ChIP-seq pre-processing

Sequencing adapters and poor quality base calls were trimmed from reads using trim_galore with default settings. Trimmed reads were aligned against the *mm10* reference genome using bowtie2 with–*sensitive* as additional option. Alignments were filtered for unmapped, multi-mapping and duplicated reads.

Signal tracks as log2 fold-change between ChIP and input were generated using deepTools bamCompare (parameters: *–scaleFactorsMethod SES –ratio log2 –bs 25 –ignoreDuplicates*) (Ramírez et al., 2016).

Peak calling was performed using MACS2 (parameters:–g *mm–p 0.001)* (Zhang et al., 2008). Publicly available datasets were re-analyzed in the same manner. ChIP-seq datasets used in this study are listed in Table S2.

#### ChIP-seq enrichment analysis

We checked if different SOM classified consensus peaks regions were enriched for TF peaks using LOLA (default settings) (Sheffield and Bock, 2016). We considered all TF peak sets with an adjusted p value < 0.01 as enriched.

To complement the *mm10* core database with TFs relevant for neural development, we added 4 newly generated samples and 39 publicly available TF ChIP-seq datasets (Table S2) Peak calls from replicate ChIP-seq experiments were considered separately.

#### RNA-seq pre-processing

RNA-seq experiments in this study were quantified using Salmon (quasi-mapping mode) with the GENCODE release M14 (Mudge and Harrow, 2015). Both single-end and paired-end reads were processed using following options (parameters: *-l A–seqBias –numBootstraps 50*). The resulting counts and transcripts per million (TPM) were used for downstream analysis.

Differential analysis of D5H versus D5SC (Gouti et al., 2014) was performed using DESeq2 with default settings. The resulting adjusted p values were used for Figure S4E.

#### Microarray analysis

Microarray data was downloaded as CEL files and imported into R. Preprocessing and normalization was done with the robust multichip average algorithm (RMA) from the oligo package (default settings) (Carvalho and Irizarry, 2010). Differential expression between two conditions was computed fitting a linear model with limma (Ritchie et al., 2015). The resulting differentially expressed genes were annotated with gene symbols using the package mouse4302.db (https://bioconductor.org/packages/release/data/annotation/html/mouse4302.db.html).

#### Interaction database

A dataset of putative gene-region interactions was downloaded from the 4DGenome database (Teng et al., 2015). The interactions were mapped to *mm9* and for further downstream analysis re-mapped to *mm10* using UCSC-liftOver with default settings. Interactions for which only one anchor could be mapped to *mm10* were removed.

Putative chromatin-chromatin interactions were mapped by filtering for anchors which overlap open chromatin sites from this study.

#### Gene ontology enrichment of CDX2 bound open chromatin sites

MNP CDX2 ChIP-seq peaks (Mazzoni et al., 2013) were overlapped with NMP, NMP-SC, spinal cord, H/SC and hindbrain regions. The resulting peak sets were used as the input for gene ontology enrichment analysis using GREAT (default settings) (McLean et al., 2010).

### Quantification and Statistical Analysis

Statistical analysis and software are described in the Figure legends and STAR Methods section.

### Data and Software Availability

The accession number for the ATAC-seq data reported in this paper is Array Express: E-MTAB-6337. The accession number for the ChIP-seq data reporter in this paper is Array Express: E-MTAB-6348.

#### Code availability

Analysis scripts are available at https://github.com/luslab/NeuralATACseq

## Supplemental Information

Table S1. Peak-to-Gene Annotation of Region-Specific Sites Identified in This Study

Table S2. List of ChIP-Seq, ATAC-Seq, mRNA-Seq, and Additional Datasets Used for Analysis

Document S1. Tables S3, S5, and S6

Table S4. List of NMP, NMP/Spinal Cord Shared, and Spinal Cord Sites and Engagement of CDX2 at These Sites
