# Supplementary material for: Nervous System Regionalization Entails Axial Allocation before Neural Differentiation
Source: Cell. 2018 Nov 1;175(4):1105–1118.e17. doi: 10.1016/j.cell.2018.09.040 (PMC6218657; doi:10.1016/j.cell.2018.09.040)
Supplement: Document S1. Tables S3, S5, and S6 [file mmc3.pdf]

TABLE S3. WNT reporter activity and CDX2 expansion in mouse embryos cultured in control versus WNT signalling conditions, Related to Figure 5.

**Stage**	**Condition**	**WNT reporter expansion**	**CDX2 expansion**
E7.0	No treatment; N=4	0/8	0/7
E7.0	FGF treatment; N=2	0/3	0/3
E7.0	CHIR treatment; N=4	6/7	7/8
E7.0	FGF/CHIR treatment; N=3	4/6	3/5
E7.5	No treatment; N=1	N/A	0/1
E7.5	FGF treatment; N=6	0/6	0/29
E7.5	CHIR treatment; N=1	N/A	0/1
E7.5	FGF/CHIR treatment; N=6	13/16	0/31

Table S5. Primers used for qPCR, Related to STAR Methods.

Primer	Sequence
Actin_F	TGGCTCCTAGCACCATGA
Actin_R	CCACCGATCCACACAGAG
Cdx2_F	TAGTCGATACATCACCATCAGG
Cdx2_R	TGATTTTCCTCTCCTTGGCTCT
Dbx1_F	CTATTTCCCAGCTTCCTCCA
Dbx1_R	GCTTCTGGAACGTCTTCTCC
Hoxb4_F	AGCACGGTAAACCCCAATTACG
Hoxb4_R	CGCGTCAGGTAGCGATTGTAG
Hoxb8_ F	CAGCTCTTTCCCTGGATG
Hoxb8_ R	CACTTCATTCTCCGATTCTG
Hoxb9_F	TAATCAAAGAGCTGGCTACG
Hoxb9_R	CCCTGGTGAGGTACATATTG
Hoxc4_F	AAGCAACCCATAGTCTACCCTTG
Hoxc4_R	CTCCGTTATAATTGGGGTTCACCGT
Hoxc6_F	CAGGTAAAGGCAAAGGGATG
Hoxc6_R	ATAGGCGGTGGAATTGAGG
Hoxc8_F	GAAGGACAAGGCCACTTAAAT
Hoxc8_R	AGGTCTGATACCGGCTGTAAGTTT
Notum_F	GCCAGTTCAAGGAAGGAGAG
Notum_R	AAGCCACTGTACCACGAACA
Olig2_F	AGACCGAGCCAACACCAG
Olig2_R	AAGCTCTCGAATGATCCTTCTTT
Pax7_F	CAGCCAGCAAGACATTCC
Pax7_R	ACCACAGATACAAGCCATTC
Phox2b_F	GATAAGGACCACTTTTGGGGC
Phox2b_R	GTTTGTATGGAACTGCGGCG
T/Bra_F	ACACACGGCTGTGAGAGGTA
T/Bra_R	TTATCATGGGACTGCAGCAT

Table S6. Primer sequences used for barcoding ATAC-seq libraries, Related to STAR Methods.

Nextera index	Primer Sequence
Ad1_noMX:	AATGATACGGCGACCACCGAGATCTACACTCGTCGGCAGCGTCAGATGTG
Ad2.1_TAAGGCGA	CAAGCAGAAGACGGCATACGAGATTCGCCTTAGTCTCGTGGGCTCGGAGATGT
Ad2.2_CGTACTAG	CAAGCAGAAGACGGCATACGAGATCTAGTACGGTCTCGTGGGCTCGGAGATGT
Ad2.3_AGGCAGAA	CAAGCAGAAGACGGCATACGAGATTTCTGCCTGTCTCGTGGGCTCGGAGATGT
Ad2.4_TCCTGAGC	CAAGCAGAAGACGGCATACGAGATGCTCAGGAGTCTCGTGGGCTCGGAGATGT
Ad2.5_GGACTCCT	CAAGCAGAAGACGGCATACGAGATAGGAGTCCGTCTCGTGGGCTCGGAGATGT
Ad2.6_TAGGCATG	CAAGCAGAAGACGGCATACGAGATCATGCCTAGTCTCGTGGGCTCGGAGATGT
Ad2.7_CTCTCTAC	CAAGCAGAAGACGGCATACGAGATGTAGAGAGGTCTCGTGGGCTCGGAGATGT
Ad2.8_CAGAGAGG	CAAGCAGAAGACGGCATACGAGATCCTCTCTGGTCTCGTGGGCTCGGAGATGT
Ad2.9_GCTACGCT	CAAGCAGAAGACGGCATACGAGATAGCGTAGCGTCTCGTGGGCTCGGAGATGT
Ad2.10_CGAGGCTG	CAAGCAGAAGACGGCATACGAGATCAGCCTCGGTCTCGTGGGCTCGGAGATGT
Ad2.11_AAGAGGCA	CAAGCAGAAGACGGCATACGAGATTGCCTCTTGTCTCGTGGGCTCGGAGATGT
Ad2.12_GTAGAGGA	CAAGCAGAAGACGGCATACGAGATTCCTCTACGTCTCGTGGGCTCGGAGATGT
